# Genotype–Phenotype Analysis of *RPGR* Variations: Reporting of 62 Chinese Families and a Literature Review

**DOI:** 10.3389/fgene.2021.600210

**Published:** 2021-06-23

**Authors:** Junxing Yang, Lin Zhou, Jiamin Ouyang, Xueshan Xiao, Wenmin Sun, Shiqiang Li, Qingjiong Zhang

**Affiliations:** ^1^State Key Laboratory of Ophthalmology, Zhongshan Ophthalmic Center, Sun Yat-sen University, Guangzhou, China; ^2^Department of Ophthalmology, West China Hospital, Sichuan University, Chengdu, China

**Keywords:** *RPGR*, retinitis pigmentosa, genotype, phenotype, exome sequencing

## Abstract

**Purpose:**

*RPGR* is the most common cause of X-linked retinitis pigmentosa (RP), of which female carriers are also frequently affected. The aim of the current study was to explore the *RPGR* variation spectrum and associated phenotype based on the data from our lab and previous studies.

**Methods:**

Variants in *RPGR* were selected from exome sequencing data of 7,092 probands with different eye conditions. The probands and their available family members underwent comprehensive ocular examinations. Similar data were collected from previous reports through searches in PubMed, Web of Science, and Google Scholar. Systematic analyses of genotypes, phenotypes and their correlations were performed.

**Results:**

A total of 46 likely pathogenic variants, including nine missense and one in-frame variants in RCC1-like domain and 36 truncation variants, in *RPGR* were detected in 62 unrelated families in our in-house cohort. In addition, a total of 585 variants, including 491 (83.9%) truncation variants, were identified from the literature. Systematic analysis of variants from our in-house dataset, literature, and gnomAD suggested that most of the pathogenic variants of *RPGR* were truncation variants while pathogenic missense and in-frame variants were enriched in the RCC1-like domain. Phenotypic variations were present between males and female carriers, including more severe refractive error but better best corrected visual acuity (BCVA) in female carriers than those in males. The male patients showed a significant reduction of BCVA with increase of age and males with exon1-14 variants presented a better BCVA than those with ORF15 variants. For female carriers, the BCVA also showed significant reduction with increase of age, but BCVA in females with exon1-14 variants was not significant difference compared with those with ORF15 variants.

**Conclusion:**

Most pathogenic variants of *RPGR* are truncations. Missense and in-frame variants located outside of the RCC1-like domain might be benign and the pathogenicity criteria for these variants should be considered with greater caution. The BCVA and refractive error are different between males and female carriers. Increase of age and location of variants in ORF15 contribute to the reduction of BCVA in males. These results are valuable for understanding genotypes and phenotypes of *RPGR.*

## Introduction

Retinitis pigmentosa (RP) is a common type of inherited retinal degenerations (IRD) characterized by impaired dark adaptation and night blindness, progressive visual field defects and pigmentary retinopathy, affecting approximately one in 3,500–4,000 people worldwide (Berger et al., 2010; Traboulsi, 2010; Sundaram et al., 2012; Zhang, 2016). RP can be inherited as an autosomal dominant, autosomal recessive, or X-linked trait, with these categories accounting for approximately 30–40%, 50–60%, and 5–15% of RP patients, respectively (Bunker et al., 1984; Grondahl, 1987; Hartong et al., 2006).

X-linked RP is one of the most severe forms of human retinal degeneration (Bird, 1975). Affected males usually suffer nyctalopia and severe and rapid progressive loss of peripheral vision with an early onset, followed by progressive central visual loss during the second to fourth decades of life, while female carriers may present a wide range of phenotypes, ranging from asymptomatic to severe phenotype (Bird, 1975; Fishman et al., 1986; Banin et al., 2007). Additionally, the phenotype of X-linked RP generally shows great phenotypic heterogeneity, including interfamily heterogeneity, in terms of the age of onset, clinical severity, rate of progression, and prevailing damage to rods and cones (Fahim et al., 2011). Variants in retinitis pigmentosa GTPase regulator (*RPGR*, OMIM 312610) account for 70–80% (Sharon et al., 2003; Pelletier et al., 2007; Shu et al., 2007) of X-linked RP cases. This protein localizes to the connecting cilium in photoreceptors and is thought to play a role in protein transport (Roepman et al., 2000; Hong et al., 2003).

In 2007, a study provided an overview of *RPGR* genotypes and the associated phenotypic variation (Shu et al., 2007). However, the widespread application of next-generation sequencing (NGS) in recent years has increased the number of variants identified in *RPGR* and expanded the known phenotypic spectrum of patients. Further comprehensive analysis of *RPGR* genotype–phenotype relationships would be expected. In addition, most of the patients previously reported to show variants in *RPGR* were recruited from America or Europe.

In this study, we performed a summary of the genotypes and corresponding phenotypes in *RPGR* from our database and the literature. The pathogenicity of the variants in *RPGR* and genotype–phenotype correlations were further assessed and summarized.

## Materials and Methods

### Samples

In an ongoing study of genetic eye diseases, we recruited 7092 probands with different eye conditions from the pediatric and Genetic Eye Clinic of the Zhongshan Ophthalmic Center, and we collected the available clinical data of the probands and their available family members with *RPGR* variations. This study was performed in accordance with the Declaration of Helsinki, and written informed consent was obtained from participating individuals or their guardians. Our study was approved by the Institutional Review Board of Zhong Shan Ophthalmic Center. All patients included in the study underwent exome sequencing [whole-exome sequencing (WES) and targeted exome sequencing (TES)]. The rare variants were defined as variants with a minor allelic frequency of less than 0.01 in general population from gnomAD database and patients with likely pathogenic variants were subsequently discriminated from the rare variants of *RPGR* in this study. Genotype–phenotype correlation was investigated by statistical analyses on different groups of patients with likely pathogenic variants of *RPGR* according to the variants in certain regions. In addition, patients with rare variants in *RPGR* were summarized based on our data (Tables 1, 2).

**TABLE 1:** 46 likely pathogenic variants in *RPGR* from 62 unrelated families (based on NM_001034853).

Variants	Exon	Nucleotide	Effect	Polyphen2	PROVEAN	REVEL	CADD	BDGP	HSF	No. of probands	Initial diagnosis	HGMD	Novel or	Evidence
		change		HVAR	pred		score			(reported)^∮^			Known	
**Missense and In-frame**														
1	2	c.124T>C	p.Cys42Arg	D	D	0.897	25.7	/	/	1	HM	NA	Novel	2, 3, 4, 5, 6
2	2	c.149T>G	p.Val50Gly	D	D	0.796	23.2	/	/	1	RP	NA	Novel	2, 3, 4, 5, 6
3	4	c.292C>A	p.His98Asn	D	D	0.84	26.6	/	/	1	CORD	NA	Novel	1, 2, 3, 4, 5, 6
4	5	c.431A>G	p.Gln144Arg	D	D	0.507	24.6	/	/	1(1)	RP	DM	Known	2, 3, 4, 5, 6
5	6	c.494G>T	p.Gly165Val	D	D	0.982	26.6	/	/	1	RP	DM	Known	1, 2, 3, 4, 5, 6
6	7	c.748T>C	p.Cys250Arg	D	D	0.906	24.5	/	/	1	RP	DM	Known	2, 3, 4, 5, 6, 7
7	8	c.878G>T	p.Arg293Met	D	D	0.295	13.92	/	/	1	RP	NA	Novel	2, 3, 4, 5, 6,
8	8	c.905G>A	p.Cys302Tyr	D	D	0.919	25.2	/	/	1	RP	DM	Known	2, 3, 4, 5, 6,
9	9	c.958G>A	p.Gly320Arg	D	D	0.959	32	/	/	1	RP	DM	Known	2, 3, 4, 5, 6,
10	10	c.1071_1073 delTGG	p.Gly358del	/	/	/	/	/	/	1(1)	RP	DM?	Known	1, 2, 4, 5, 6,
**Truncation**														
1	2	c.140_144dup CTGCT	p.Ser47Phefs*23	/	/	/	/	/	/	1(1)	RP	NA	Known	1, 2, 4, 5, 6
2	6	c.473del	p.Asp158Glufs*17	/	/	/	/	/	/	1(1)	RP	NA	Known	2, 4, 5, 6
3	6	c.530dupT	p.Ser178Lysfs*2	/	/	/	/	/	/	1(1)	RP	DM	Known	1, 2, 4, 5, 6
4	10	c.1243_1244del	p.Arg415Glyfs*37	/	/	/	/	/	/	1	RP	DM	Known	2, 4, 5, 6
5	14	c.1685_1686del	p.His562Argfs*20	/	/	/	/	/	/	1(1)	RP	DM	Known	1, 2, 4, 5, 6
6	ORF15	c.1872_1873del	p.Glu624Aspfs*5	/	/	/	/	/	/	1	RP	NA	Known	1, 2, 4, 5, 6
7	ORF15	c.2075dupG	p.Glu693Argfs*77	/	/	/	/	/	/	1(1)	RP	NA	Known	2, 4, 5, 6
8	ORF15	c.2190del	p.Glu732Argfs*83	/	/	/	/	/	/	1	RP	NA	Novel	1, 2, 4, 5, 6
9	ORF15	c.2236_2237del	p.Glu746Argfs*23	/	/	/	/	/	/	6(2)	RP	NA	Known	1, 2, 4, 5, 6
10	ORF15	c.2272del	p.Glu758Lysfs*57	/	/	/	/	/	/	1	RP	NA	Novel	1, 2, 4, 5, 6
11	ORF15	c.2384del	p.Glu795Glyfs*20	/	/	/	/	/	/	1	RP	NA	Known	2, 4, 5, 6
12	ORF15	c.2403_2406del	p.Glu802Glyfs*12	/	/	/	/	/	/	1(1)	RP	NA	Known	2, 4, 5, 6
13	ORF15	c.2405_2406del	p.Glu802Glyfs*32	/	/	/	/	/	/	5(4)	HM, RP	NA	Known	1, 2, 4, 5, 6
14	ORF15	c.2420_2435del	p.Glu807Glyfs*3	/	/	/	/	/	/	1(1)	RP	NA	Known	2, 4, 5, 6
15	ORF15	c.2442_2445del	p.Gly817Lysfs*2	/	/	/	/	/	/	2	MD, RP	NA	Known	2, 4, 5, 6
16	ORF15	c.2476_2477del	p.Arg826Glyfs*8	/	/	/	/	/	/	2(2)	HM, RP	NA	Known	1, 2, 4, 5, 6
17	ORF15	c.3027_3028del	p.Glu1010Glyfs*68	/	/	/	/	/	/	3(1)	HM, RP	NA	Known	1, 2, 4, 5, 6
18	ORF15	c.3092del	p.Glu1031Glyfs*58	/	/	/	/	/	/	1	HM	NA	Known	1, 2, 4, 5, 6
19	ORF15	c.3096_3097del	p.Glu1033Argfs*45	/	/	/	/	/	/	2	HM	NA	Known	1, 2, 4, 5, 6
20	ORF15	c.3241del	p.Asp1081Metfs*8	/	/	/	/	/	/	1(1)	HM	NA	Known	1, 2, 4, 5, 6
21	ORF15	c.3317del	p.Lys1106Serfs*25	/	/	/	/	/	/	1	RP	NA	Known	2, 4, 5, 6
22	ORF15	c.3364del	p.Met1122Cysfs*9	/	/	/	/	/	/	1(1)	HM	NA	Known	1, 2, 4, 5, 6
23	2	c.93G>A	p.Trp31*	/	/	/	33	/	/	1	RP	DM?	Known	1, 2, 4, 5, 6
24	2	c.122C>G	p.Ser41*	/	/	/	35	/	/	1(1)	RP	DM	Known	1, 2, 4, 5, 6
25	3	c.191G>A	p.Trp64*	/	/	/	39	/	/	1	RP	NA	Novel	2, 4, 5, 6
26	5	c.352C>T	p.Gln118*	/	/	/	33	/	/	2(1)	RP	DM	Known	1, 2, 4, 5, 6, 7
27	10	c.1234C>T	p.Arg412*	/	/	/	34	/	/	2(1)	RP	DM	Known	1, 2, 4, 5, 6
28	11	c.1345C>T	p.Arg449*	/	/	/	23.7	/	/	1	RP	DM	Known	1, 2, 4, 5, 6
29	13	c.1561C>T	p.Gln521*	/	/	/	35	/	/	1	RP	NA	Known	1, 2, 4, 5, 6
30	ORF15	c.2248G>T	p.Glu750*	/	/	/	24.9	/	/	1	RP	NA	Novel	2, 4, 5, 6
31	ORF15	c.2491G>T	p.Glu831*	/	/	/	32	/	/	1	RP	NA	Known	2, 4, 5, 6
32	IVS4	c.310 + 1G>A	/	/	/	/	33	SD	SD	1(1)	RP	DM	Known	2, 4, 5, 6
33	IVS9	c.1060−1G>A	/	/	/	/	33	SA	SA	1	RP	NA	Novel	2, 4, 5, 6
34	IVS12	c.1506 + 1G>T	/	/	/	/	33	SD	SD	1	HM	NA	Novel	2, 4, 5, 6
35	IVS12	c.1506 + 2T>C	/	/	/	/	32	SD	SD	1	RP	NA	Novel	2, 4, 5, 6
36	IVS13	c.1573−2A>G	/	/	/	/	28.2	NSC	SA	1	RP	DM	Known	2, 4, 5, 6

CORD, cone-rod dystrophy; DM, disease-causing mutations; HM, high myopia; MD, macular degeneration; NA, not available; NSC, no splicing change; RP, retinitis pigmentosa; SA, splicing acceptor; SD, splicing donor; *, termination codon; /, not applicable. BDGP, Berkeley Drosophila Genome Project; HGMD, the Human Gene Mutation Database; HSF, Human Splicing Finder; ()∮ : Previously reported by our lab. None of the variants were recorded in gnomAD except c.3027_3028delGG. Evidence that variant is likely pathogenic: 1 = segregate with inherited eye diseases in one or more families (males with variants were affected); 2 = variants identified in one or more families with eye disease accompany with RP, CORD, COD, MD, or HM; 3 = at least three of four predicting tools are pathogenic; 4 = MAF ≤ 4.7 × 10^–5^ or absence in gnomAD database; 5 = other known IRD pathogenic variants were not identified; 6 = variants does not find in controls; 7 = variants are de novel.

**TABLE 2:** 51 benign or likely benign variants in *RPGR* from 101 unrelated families (based on NM_001034853).

Variants	Exon	Nucleotide	Effect	➀	➁	➂	➃		gnomAD			No. of probands	Diagnosis	Novel or	Evidence
	
		change						AF	Hemi	EA	Hemi	(reported)^∮^		Known	
**Missense**															
1	ORF15	c.1910G>A	p.S637N	B	N	0.055	23.6	NA	NA	NA	NA	1(1)	HM	Known	3
2	ORF15	c.1930G>A	p.V644M	B	N	0.004	9.241	NA	NA	NA	NA	1	RP	Novel	1, 3, 5
3	ORF15	c.1957G>A	p.G653S	B	N	0.019	10.57	NA	NA	NA	NA	1	BCD	Novel	2, 3, 5
4	ORF15	c.1967A>T	p.D656V	B	N	0.043	16.41	NA	NA	NA	NA	1(1)	HM	Known	3
5	ORF15	c.2135A>G	p.Q712R	B	N	0.038	0.077	9/157035	2	9/12249	2	8(1)	N, HM, RB, ONH, G, HYP	Known	2, 3, 4, 5, 6
6	ORF15	c.2200G>A	p.E734K	P	N	0.04	18.53	6/117024	1	5/8773	1	5(1)	G, NYS, HM	Known	2, 3, 4, 5
7	ORF15	c.2342C>T	p.A781V	B	N	0.082	5.756	11/126393	4	0/9010	0	1	RP	Novel	3, 4
8	ORF15	c.2357A>C	p.K786T	B	N	0.013	13.68	NA	NA	NA	NA	1	Best	Novel	2, 3, 5
9	ORF15	c.2606A>G	p.E869G	B	N	0.062	14.86	25/40040	0	0/3847	0	1	RP	Novel	3, 4
10	ORF15	c.2995G>T	p.G999W	P	N	0.025	17.62	1/71397	0	1/7897	0	1	RP	Novel	3, 4, 5
11	ORF15	c.3035A>G	p.E1012G	B	N	0.031	12.1	5/110513	2	5/9144	2	2	RB, RP	Novel	2, 3, 4, 5
12	ORF15	c.3088G>A	p.G1030R	B	N	0.046	15.2	NA	NA	NA	NA	1	RP	Novel	1, 3, 5
13	ORF15	c.3122A>G	p.E1041G	B	N	0.04	14.35	NA	NA	NA	NA	2	LCA, HM	Novel	1, 3
14	ORF15	c.3220G>A	p.E1074K	B	N	0.034	14.59	3/181654	1	2/13859	1	1	G	Novel	2, 3, 4
15	ORF15	c.3271A>T	p.I1091L	B	N	0.006	10.61	NA	NA	NA	NA	2	N, RB	Novel	2, 3, 6
16	ORF15	c.3439C>G	p.H1147D	P	N	0.257	24.1	NA	NA	NA	NA	1	RP	Novel	3
**In-frame**															
1	ORF15	c.2360_2362del	p.G787del	/	/	/	/	3/127259	0	3/9008	0	1	HM	Novel	4
2	ORF15	c.2447_2461del	p.G816_E820del	/	/	/	/	123/103374	16	2/7866	1	4(1)	CORD, RP, RB, HM	Known	2, 4
3	ORF15	c.2952_2954del	p.E985del	/	/	/	/	2/52677	1	1/6728	0	1	LCA	Novel	4
4	ORF15	c.3032_3043del	p.G1011_E1014del	/	/	/	/	3/116321	0	1/9309	0	1	HM	Novel	4
5	ORF15	c.3051_3053del	p.E1018del	/	/	/	/	547/119184	118	1/9395	1	1	NYS	Novel	4
6	ORF15	c.3105_3122delins^†^	p.E1037_E1041delins^#^	/	/	/	/	NA	NA	NA	NA	7	N, HM, RD, FEVR, RP	Novel	1, 2, 6
7	ORF15	c.3123_3125del	p.E1042del	/	/	/	/	25/191580	6	2/14266	0	2(1)	RP	Known	4
8	ORF15	c.3133_3135del	p.E1045del	/	/	/	/	4/176793	3	0/13596	0	1	RP	Novel	NA
9	ORF15	c.3170_3172del	p.R1057del	/	/	/	/	10/201586	5	1/14789	0	1	RP	Novel	4
10	ORF15	c.3180_3182del	p.E1066del	/	/	/	/	2/181016	0	0/13852	0	1	HM	Novel	NA
11	ORF15	c.3189_3191del	p.E1066del	/	/	/	/	2/181292	0	1/13854	0	1	G	Novel	2, 4
12	ORF15	c.3195_3197del	p.E1066del	/	/	/	/	2/181292	0	1/13854	0	1	HM	Novel	4
13	ORF15	c.3225_3227del	p.E1076del	/	/	/	/	1/181925	0	0/13860	0	1	HM	Novel	NA
**Exon1-14**															
1	1	c.7G>A	p.E3K	P	N	0.127	22.2	NA	NA	NA	NA	1	G	Novel	2, 3
2	2	c.37G>A	p.A13T	D	D	0.485	25.3	NA	NA	NA	NA	1	FEVR	Novel	2
3	2	c.112G>A	p.V38I	B	N	0.051	0.066	NA	NA	NA	NA	1	LD	Novel	2, 3, 5
4	4	c.277G>T	p.A93S	D	D	0.59	24.4	1/182876	1	1/13846	1	4	RD, HM, COD, CD	Novel	1, 2, 4, 5
5	6	c.522A>T	p.L174F	D	D	0.55	15.94	NA	NA	NA	NA	1	N	Novel	6
6	7	c.738C>G	p.I246M	P	N	0.404	17.84	NA	NA	NA	NA	1	HM	Novel	3
7	8	c.782A>C	p.N261T	B	N	0.19	7.487	4/180617	2	4/13651	2	4	N, RP, RD, HM	Novel	2, 3, 4, 5, 6
8	7	c.727G>A	p.E243K	P	N	0.33	23.1	NA	NA	NA	NA	1	G	Novel	2
9	10	c.1163C>T	p.A388V	B	N	0.049	5.981	44/183213	14	0/13847	0	2	G, HM	Novel	2, 3, 4
10	11	c.1270A>G	p.M424V	B	D	0.12	6.942	3/182944	0	0/13818	0	1	PHPV	Novel	2, 3
11	11	c.1282C>G	p.L428V	P	N	0.129	14.6	35/204797	14	35/14807	14	8(1)	G, HM, OA, RP, RD, RRD	Known	2, 3, 4, 5
12	11	c.1331A>G	p.N444S	B	N	0.012	0.002	2/183351	0	0/13858	0	1	RRD	Novel	2, 3, 5
13	11	c.1367A>G	p.Q456R	B	N	0.016	0.052	1704/205075	594	0/14852	0	1	G	known	2, 3, 4
14	13	c.1519A>G	p.S507G	P	D	0.046	22	4/164448	0	4/12070	0	2	LCA, G	Novel	1, 2, 4
15	13	c.1561C>G	p.Q521E	B	N	0.046	16.13	5/171108	3	5/12659	3	6	G, MC, RB, HYP, HM	Novel	1, 2, 3, 4
16	14	c.1585A>G	p.I529V	B	N	0.006	0.002	2/181278	0	1/13849	0	3	NYS, HM, LCA	Novel	2, 3
17	14	c.1622A>G	p.N541S	B	N	0.016	0.343	NA	NA	NA	NA	1	RRD	Novel	2, 3, 5
18	14	c.1628A>G	p.D543G	B	N	0.011	4.167	NA	NA	NA	NA	1	RP	Novel	3
19	14	c.1630A>G	p.S544G	P	N	0.049	19.15	2/182957	1	1/13858	0	3(1)	N, G, HM	Known	2, 3, 4, 6
20	14	c.1720A>G	p.T574A	B	N	0.013	0.527	NA	NA	NA	NA	1	LCA	Novel	3, 5
21	14	c.1721C>T	p.T574M	B	N	0.008	1.128	23/204508	9	1/14838	0	1	HM	Novel	3, 4
22	10	c.1117_1119dupAAA	p.K373dup	/	/	/	/	12/182610	6	12/13812	6	3	MD, HM, RB	Novel	1, 2, 4

*AF, allele frequency; CD, corneal degeneration; COD, cone dystrophy; CORD, cone-rod dystrophy; EA, East Asians; G, glaucoma; HM, high myopia; Hyp, Hypermetropia; LCA, Leber congenital amaurosis; LD, lens dislocation; MC, macular coloboma; MD, macular degeneration; N, normal; NA, not available; NYS, nystagmus; OA, ocular albinism; ONH, Optic nerve hypoplasia; PHPV, Persistent Hyperplastic Primary Vitreous; RB, retinoblastoma; RD, retinal diseases; RP, retinitis pigmentosa; RRD, Rhegmatogenous Retinal Detachment; /, not applicable; FEVR, familial exudative vitreoretinopathy; ^†^AGAAAGGGAAAAGGAGGG; ^#^ArgGluLysGluGly; ()∮ Previously reported by our lab. 1, polyphen-2 HVAR; 2, PROVEAN; 3, REVEL; 4, CADD. gnomAD, genome aggregation database; HGMD, the Human Gene Mutation Database.*

*Evidence that variant is benign or likely benign: 1 = does not segregate with inherited eye diseases in one or more families (males with variants were unaffected); 2 = variant identified in one or more families with eye disease other than RP, CORD, COD, MD, or HM; 3 = at least two of four predicting tools are benign; 4 = MAF ≥ 4.7 × 10^–5^ in gnomAD database; 5 = identified other known IRD pathogenic variants; 6 = verified in controls.*

*All variants are not recorded in HGMD except the variant c.1630A>G*.

### Exome Sequencing

Exome sequencing, including WES and TES, was conducted in the patients included in our study. Whole-exome sequencing was performed on 5,307 probands using a commercial service as described in our previous study (Li et al., 2015). Genomic DNA from the probands was sheared and fragments of an approximate 150 bp were selected. Exome was captured by an Agilent SureSelect Human All Exon Enrichment Kit (Agilent, Santa Clara, CA, United States). Library quality was assessed using an Agilent 2100 Bioanalyzer and were then sequenced on the Illumina HiSeq platform (Illumina, San Diego, CA, United States) with an average depth of at least 125-fold. After filtering out low quality reads, and remaining clean data was verified by aligning the sequencing with the UCSC hg19^1^ reference using the Burrows-Wheeler Aligner (BWA^2^). Variants were detected by SAMTOOLS^3^ and were annotated and predicted by SnpEff^4^, ANNOVAR^5^, and dbNSFP^6^, respectively.

Targeted-exome sequencing was conducted on 1,785 probands by our lab as described in our previous study (Wang et al., 2019). Approximately 200 bp fragments were obtained from genomic DNA using a Bioruptor Plus (Diagenode, Liege, Belgium). A paired-end library was prepared using a KAPA HTP Library Preparation kit (Roche, Basel, Switzerland). Targeted exome was captured using a designed NimbleGen SeqCap EZ Prime Choice kit (Roche, Basel, Switzerland). Library quality was assessed using an Agilent 2100 Bioanalyzer and were then sequenced on an Illumina Nextseq550 Analyzer using the Illumina NextSeq550 Mild output v2 kit (150 PE) (Illumina, San Diego, CA, United States) with an average depth of 250-fold. Variant calling and annotation were analyzed using the StrandNGS software (Karnataka, India) according to the manufacturer’s instructions. The UCSC Genome Browser on Human hg19 Assembly was used as an alignment reference. The dbNSFP was used for predictions of missense variants. The list of 126 target genes, including *RPGR*, in TES has been described in our previous study (Wang et al., 2019). Variants in *RPGR* identified through WES and TES were selected and filtered via multistep bioinformatics analyses as previously reported (Xu et al., 2014; Li et al., 2015; Sun et al., 2015; Zhou L. et al., 2018). Additionally, we used CADD^7^ and REVEL^8^ to further predict the severity of the missense variants in *RPGR*. Data from the Genome Aggregation Database (gnomAD^9^) and Human Genome Mutation Database (HGMD^10^) were included as references for evaluating the pathogenicity of the variants in *RPGR*. Selected remaining variants were verified by Sanger sequencing. The pedigrees and sequence diagrams of potential likely pathogenic variants are shown in Supplementary Figures 1, 2.

### Phenotype Analysis in Our Lab

Probands and available family members with variants in *RPGR* were recruited for further comprehensive ocular examinations. All of the examinations were performed by the same experienced team of ophthalmologists. A detailed family and ophthalmic history were obtained. The comprehensive ocular examinations included best corrected visual acuity (BCVA), refractive error (RE), and spectral domain-optical coherence tomography (SD-OCT).

Refractive error was measured using an autorefractometer (Topcon KR-8000, Paramus, NJ, United States) after mydriasis with tropicamide (Mydrin-P, Santen Pharmaceutical, Japan). An optical biometer (IOL master V5.0, Carl Zeiss Meditec AG, Germany) was used to detect the ocular biometric axial length. Full-field electroretinogram (ERG) responses were recorded in patients in accordance with the standards of the International Society for Clinical Electrophysiology of Vision for evaluating retinal disorders, using an Espion ERG system (Diagnosys LLC, United States). Optical coherence tomography of the macular and optic disks was performed via SD-OCT (Optovue, Inc., United States).

### Literature Review of *RPGR* Variants and Ophthalmologic Outcomes

The variants and clinical data of patients with *RPGR* were obtained by searching the literature for the keyword *RPGR* in three databases: PubMed^11^, Web of Science^12^, and Google Scholar^13^ (Meindl et al., 1996; Roepman et al., 1996; Andreasson et al., 1997, 2003; Buraczynska et al., 1997; Fujita et al., 1997; Jacobson et al., 1997; Weleber et al., 1997; Bauer et al., 1998; Fishman et al., 1998a,b; Miano et al., 1998, 1999; Dry et al., 1999; Flaxel et al., 1999; Rosenberg et al., 1999; Zito et al., 1999, 2000, 2003; Liu et al., 2000, 2002; Vervoort et al., 2000; Guevara-Fujita et al., 2001; Yokoyama et al., 2001; Zhao et al., 2001, 2020; Aguirre et al., 2002; Ayyagari et al., 2002; Breuer et al., 2002; Demirci et al., 2002, 2004, 2005, 2006; Pusch et al., 2002; Rozet et al., 2002; Yang et al., 2002, 2014; Bader et al., 2003; Barnes et al., 2003; Iannaccone et al., 2003, 2008; Koenekoop et al., 2003; Lorenz et al., 2003; Rebello et al., 2003; Sharon et al., 2003; Wegscheider et al., 2004; Adamian et al., 2005; Ebenezer et al., 2005; Jin et al., 2005, 2006, 2007a,b, 2008; Wang et al., 2005, 2015; Chakarova et al., 2006; Garcia-Hoyos et al., 2006; Moore et al., 2006; Sullivan et al., 2006, 2013; Aleman et al., 2007; Banin et al., 2007; Chang et al., 2007; Duncan et al., 2007; Neidhardt et al., 2007, 2008; Pelletier et al., 2007; Prokisch et al., 2007; Sandberg et al., 2007; Shu et al., 2007; Walia et al., 2008; Al-Maskari et al., 2009; Ruddle et al., 2009; Ji et al., 2010; Sheng et al., 2010; Wu et al., 2010; Bowne et al., 2011; Fahim et al., 2011, 2020; Glaus et al., 2011; Li N. et al., 2011; Li Z.L. et al., 2011; Liskova et al., 2011; Thiadens et al., 2011; Branham et al., 2012, 2018; O’Sullivan et al., 2012; Acton et al., 2013; Bukowy-Bieryllo et al., 2013; Churchill et al., 2013; Eisenberger et al., 2013; Huang et al., 2013, 2014, 2015a,b, 2019; Kousal et al., 2013, 2014; Liu and Zack, 2013; Pyo Park et al., 2013; Zahid et al., 2013; Glockle et al., 2014; Gonzalez-del Pozo et al., 2014; Hu et al., 2014; Oishi et al., 2014; Pierrottet et al., 2014; Wang F. et al., 2014; Wang J. et al., 2014; Xu et al., 2014, 2019; Almoguera et al., 2015; Chassine et al., 2015; Consugar et al., 2015; Fernandez-San Jose et al., 2015; Ge et al., 2015; Kastner et al., 2015; Ogino et al., 2015; Sharon and Banin, 2015; Sun et al., 2015; Haddad et al., 2016; Li et al., 2016; Parmeggiani et al., 2016; Tiwari et al., 2016; Bellingrath et al., 2017; Hendriks et al., 2017; Kalitzeos et al., 2017; Stone et al., 2017; Tee et al., 2017; Birtel et al., 2018a,b; Chiang et al., 2018; Nanda et al., 2018; Talib et al., 2018, 2019; Wawrocka et al., 2018; Zhou L. et al., 2018; Zhou Q. et al., 2018; Gill et al., 2019; Koyanagi et al., 2019; Mawatari et al., 2019, 2020; Sanchez Tocino et al., 2019; Tang et al., 2019; Zhang Z. et al., 2019; Dan et al., 2020; Foote et al., 2020; Menghini et al., 2020; Nguyen et al., 2020; Rodriguez-Munoz et al., 2020; Salvetti et al., 2020; Zampaglione et al., 2020) on July 01, 2020. The papers were limited to English-language reports of definitive variants in *RPGR*. Variant descriptions based on the work of Meindl et al. (1996) were converted to descriptions based on NM_001034853. Variants in *RPGR* previously reported to be likely pathogenic were summarized in Supplementary Table 1 based on the literature.

Clinical data were collected to perform further comparisons between genders, ages, locations and variation types. Spherical equivalent refraction (SER) was calculated by adding spherical refraction to half the cylindrical refraction.

### Statistical Analysis

Analyses were performed using R software and SPSS version 23. Logistic regression was used to screen out the factors influencing BCVA in males and females. Median (IQR, interquartile range) were used for continuous parameters. Mann–Whitney *U* test was used to compare the BCVA and refractive error among groups, namely (1) patients with variants in exon1-14; (2) patients with variants in ORF15; (3) patients with variants in RCC1-like domain; (4) patients with missense and in-frame variants; (5) patients with truncation variants. The corrected significant *P*-value for this study should be less than 0.017 (α = 0.05/3) according to the Bonferroni method.

## Results

### Identification of *RPGR* Variants in 7,092 Probands With Different Eye Conditions in Our Lab

A total of 121 variants, including 15 polymorphisms, eight 3′UTR variants, one synonymous variant and 97 rare variants, were detected in 7,092 probands. Of the 97 rare variants, 46 likely pathogenic variants (11 novels) and 51 likely benign variants were identified. Among the 46 likely pathogenic variants, nine missense variants, one in-frame variant and 17 truncation variants were located in exon1-14, and the remaining 19 truncation variants were located in ORF15 (Table 1). The 46 likely pathogenic variants were identified in 62 families, of which truncation variants were identified in 52 (83.9%, 52/62), while missense and in-frame variants were identified in nine (14.5%, 9/62) and one (1.6%, 1/62) family, respectively. Of the other 51 likely benign variants, 21 missense variants and one in-frame variant, were identified in exon1-14, while 16 missense and 13 in-frame variants were detected in ORF15 (Table 2).

### Review of *RPGR* Genotypes From Our Lab and Previous Literature

A total of 585 variants have been reported in previous literature, including 491 truncations, 84 missenses, and 10 in-frame variants. Of the 94 missense and in-frame variants, 81 were located in the RCC1-like domain, while the remaining 13 were located outside the domain (Supplementary Table 1). A total of 585 previously reported variants, combining 46 likely pathogenic variants with our laboratory data, a total of 606 variants were analyzed (25 variants were repetitive).

#### Pathogenicity Evaluation of Missense and In-Frame Variants Located Outside of the RCC1

**FIGURE 1:**
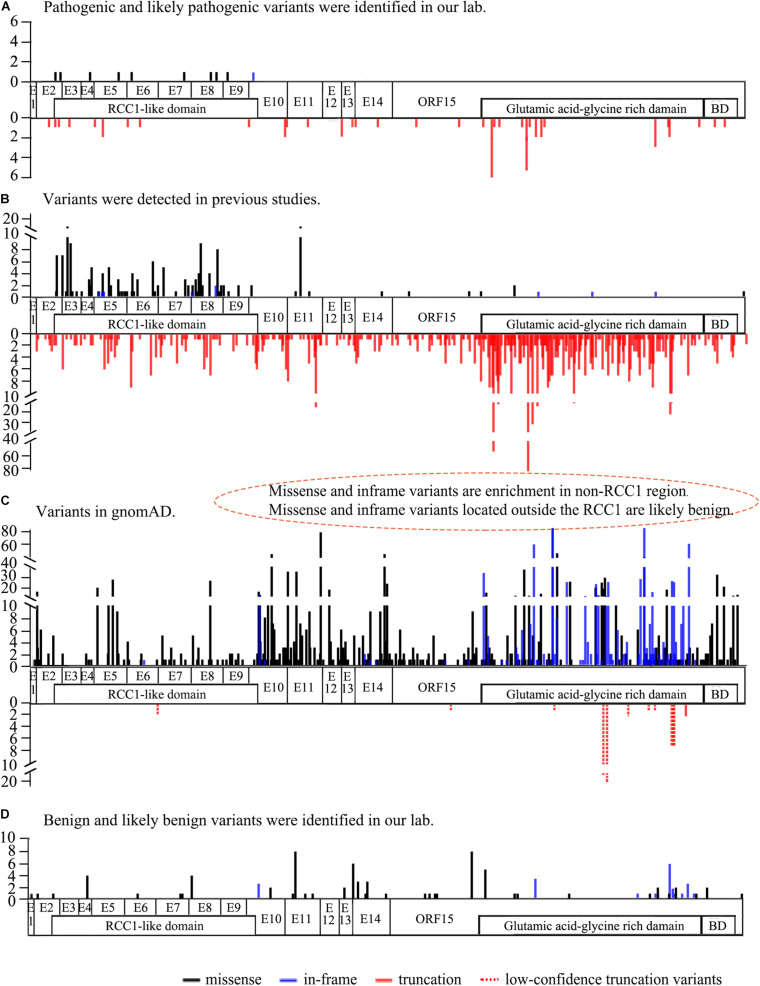
The frequency and location of the variants from our lab, previous studies, and the gnomAD database (Ref. NM_001034853). **(A)** The frequency and location of pathogenic and likely pathogenic *RPGR* variants detected in our lab. Missense and in-frame variants are distributed above the structure, and truncation variants are shown below the structure. **(B)** The frequency and location of *RPGR* variants identified in previous studies. Missense and in-frame variants enriched in the RCC1-like domain are shown above the structure, and truncation variants are indicated below the structure. Gross deletion variants are not shown here. **(C)** The frequency and location of *RPGR* variants from the gnomAD database. Missense and in-frame variants are significantly enriched in the non-RCC1-like domain above the structure. Truncation variants in all coding regions below the structure. Of the 11 truncation variants, 10 were low confidence truncations (dotted line). **(D)** The frequency and location of benign and likely benign *RPGR* variants identified in our lab. The white regions represent the coding regions. RCC1-like domain: p.38∼367, BD: basic domain p.1086-1139, Glutamic acid-glycine-rich domain: p.728∼1084.

A total of 57 missense and in-frame variants were located outside of the RCC1 region, including 45 variants from our in-house cohort and 13 from literature were identified (one variant was repetitive) (Table 2 and Supplementary Table 1). The following lines of evidence suggested that these variants in *RPGR* might not be disease causing. (1) Missense and in-frame variants were significantly enriched outside of the RCC1 region according to the gnomAD database, and the frequency was obviously high (Figure 1). (2) Most of these variants were identified in one or more probands with different eye conditions other than RP or closely relative early onset high myopia (HM), cone-rod dystrophy (CORD), cone-dystrophy (COD), or macular degeneration (MD) (Table 2). (3) All but two missense variants (c.37G > A and c.1519A > G) located outside of the RCC1 were predicted to be benign by at least two of four prediction tools (90% cutoff score: 0.29 in REVEL and 21.5 in CADD) (Table 2). (4) A few patients showed variants in other known IRD genes, and some variants were verified in unaffected controls. (5) Segregation analysis contributed further evidence that missense and in-frame variants in non-RCC1 regions are not disease causing, and the corresponding pedigrees are shown in Supplementary Figure 3. (6) A previous study reported frequent in-frame deletions of 3–36 bp in healthy controls, suggesting that in-frame variants are benign (Karra et al., 2006). In addition, Zhang Q. et al. (2019) developed an *in vitro* assay illustrating that some variations located outside of the RCC1 region might be non-disease-causing polymorphisms.

### *RPGR*-Associated Phenotype Analysis of Based on Our Data and the Literature

#### BCVA in Patients With *RPGR* Variations

**FIGURE 2:**
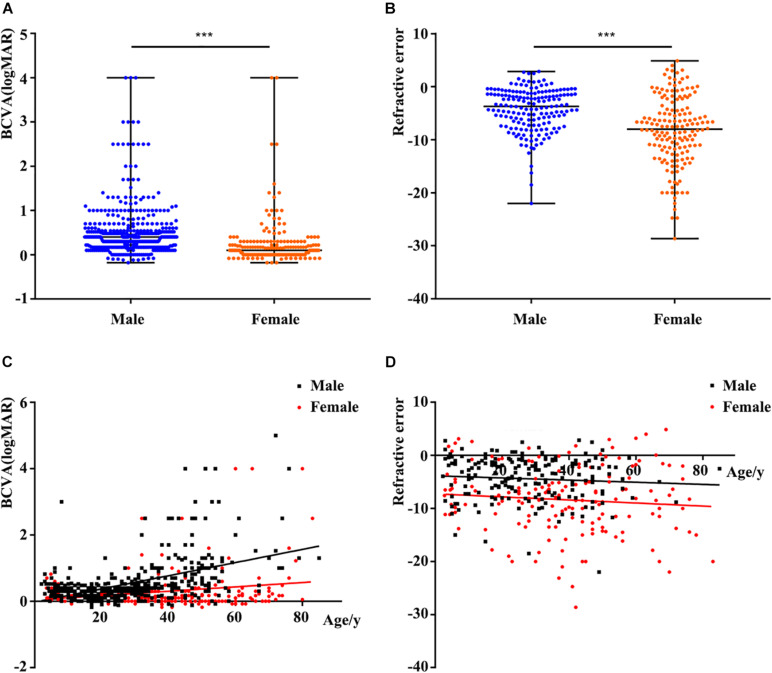
Comparison of phenotypes according to different factors. **(A)** Comparison of logMAR BCVA between males and females. The BCVA of female carriers was better than that of male patients. **(B)** Comparison of refractive error (RE) between males and females. Spherical equivalent refraction represents the severity of RE. The RE of female carriers was more serious than that of males. **(C)** Scatterplots of logMAR BCVA and age, the two fitted lines correspond to male (black) and female (red) patients. A significant reduction of BCVA with increase of age in both males and females. **(D)** Scatterplots of RE and age, the two fitted lines correspond to male (black) and female (red) patients. The trends of the two lines are basically smooth. BCVA, best corrected visual acuity. ^∗∗∗^, *P* value less than 0.001.

**FIGURE 3:**
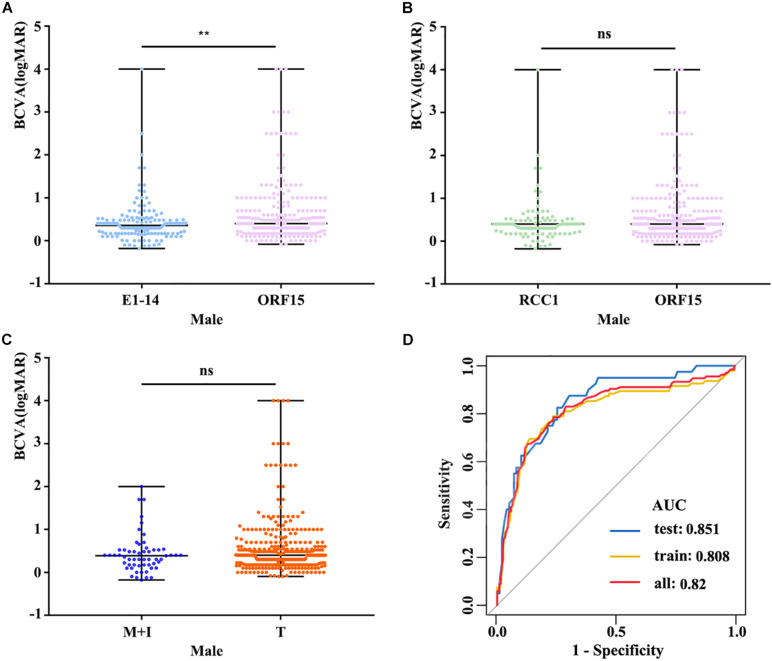
**(A)** The logMAR BCVA of male patients with variants in exon1-14 and ORF15 showed that patients with variants in exon1-14 have a better visual acuity. **(B)** Patients with variants in RCC1-like domain were no significant difference compared to those in ORF15. **(C)** Comparison of logMAR BCVA between M + I and T, there was no significant difference in variation type. **(D)** ROC curves suggested that our model shows high sensitivity and specificity in distinguishing different degrees of BCVA. The datasets used for AUC analysis were from available males’ data and were randomly divided into two independent datasets (training and test datasets) by the R-software. BCVA, best corrected visual acuity; E1-14, exon1-exon14; RCC1, RCC1-like domain; M + I, missense and in-frame; T, truncation. ns, no statistical significance; ^∗∗^, *P* value less than 0.01.

The clinical data of the probands and available families with pathogenic variants from our database and previous studies are summarized in Supplementary Tables 2, 3. The statistical results table were shown in Supplementary Table 4. BCVA showed a significant reduction with increase of age in both males and females (*r* = 0.479 and *r* = 0.216, respectively) (Figure 2C). Better BCVA in female carriers (0.10 [0.00, 0.30] logMAR) than in male patients (0.40 [0.17, 0.60] logMAR) (*P* = 7.41E-25) (Figure 2A). Logistic regression was used to screen out the factors influencing BCVA in males and females, and the receiver operating characteristic (ROC) curves suggested that our model showed high sensitivity and specificity in distinguishing the different degrees of BCVA (Figure 3D). For males, the variation type was not associated with BCVA (*P* = 0.183) (Figure 3C). The BCVA of male patients with variants in exon1-14 (0.36 [0.17, 0.48] logMAR) was significantly better than that of male patients with variants in ORF15 (0.40 [0.20, 0.70] logMAR) (*P* = 0.005) after age adjustment, however, the comparison between RCC1 and ORF15 was no significant difference (*P* = 0.048) (Figures 3A,B). BCVA was not associated with location or variation type in female carriers (all *P* > 0.05, respectively) (Supplementary Figures 5A–C).

#### Refractive Error in Patients With *RPGR* Variations

Spherical equivalent refraction was used to assess the severity of the RE. The percentage of female carriers with high myopia was significantly greater than that of males (109/165 and 51/179, respectively). Females with variants in *RPGR* showed a more serious of SER than males (−8.00 [−12.00, −4.19] in female carriers and −3.72 [−6.99, −1.28] in male patients, *P* = 5.46E-10) (Figure 2B). Logistic regression showed that RE was unrelated to age, location or variation type in both male patients and female carriers (all *P* > 0.05) (Figure 2D and Supplementary Figures 4, 5D–F).

In addition, the fundus changes vary widely among patients with *RPGR* variants, including gray-white fundal spots, tessellated fundus, retinal degeneration to macular degeneration in males and female carriers.

## Discussion

In this study, 97 rare RPGR variations were detected in our in-house exome sequence data. A total of 585 variants were identified from previous studies. All in-house data and previous literature data were combined for further genotype–phenotype analysis.

Enrichment and the frequency analyses showed that the benign variants were enriched in non-RCC1 regions. Multistep bioinformatics analyses provided evidence that the corresponding prediction scores were lower than those of variants in the RCC1 region. In addition, segregation and phenotypic consistency analyses further confirmed the benign nature of the variants. A few families also showed variants in other known IRD genes, and some variants were verified in unaffected controls. In previous studies, three families with compound heterozygous variants in *RPGR*, one allele was an in-frame variant in ORF15, and the other allele was a truncation variant (Pelletier et al., 2007; Neidhardt et al., 2008). Moreover, in-frame variants in ORF15 (spanning 3–36 bp) in healthy individuals were reported in a previous study, suggesting that at least some in-frame variants in ORF15 of *RPGR* might not be causative (Karra et al., 2006). An *in vitro* assay developed in a previous study illustrated that some variations located outside of the RCC1 regions might be non-disease-causing polymorphisms (Zhang Q. et al., 2019). Taken together, these findings suggest that at least some missense changes and in-frame variants in the non-RCC1 region might not be pathogenic. Interestingly, several truncation variants at C-terminal region of *RPGR* had a high frequency in the gnomAD database, but all of them were low-confidence. If the high frequency of these truncations were validated, the pathogenicity of truncations around and downstream of these variants should be considered with greater caution.

More than 85% of the patients with pathogenic RPGR variants had RP. The remainder were diagnosed with a variety of X-linked retinal diseases, including IRD, CORD, COD, high myopia, and MD, among others. The BCVA of the probands with *RPGR* was age depended, and the BCVA of female carriers was better than that of male patients. In addition to age, the location of the variants in *RPGR* might play important roles in male patients with BCVA but not in female patients. Male patients with variants in exon1-14 retained better BCVA.

Based on our analysis, there were no significant differences in the SER with regard to the variation type, location or age in either males or females. These results suggest that progression of myopia is relatively slow in patients with variants in *RPGR*. Because some probands exhibited high myopia in the early stage, the specific screening of *RPGR* was initially not carried out in many of these patients. This emphasizes the importance of performing a comprehensive examination of patients with early-onset high myopia and of considering the possibility that *RPGR* variants may exist in these patients. RE was only associated with gender and was more serious in females than in males.

In summary, the results of this study suggested that missense and in-frame variants located outside the RCC1-like domain are likely benign. The pathogenicity criteria for *RPGR* should be considered with greater caution. Increase of age and location of variants in ORF15 contribute to the reduction of BCVA in males. These results are valuable for understanding genotypes and phenotypes of *RPGR*.

## Data Availability Statement

The data supporting the conclusions of this article will be made available by the authors, without undue reservation.

## Ethics Statement

The studies involving human participants were reviewed and approved by Institutional Review Board of Zhong Shan Ophthalmic Center. Written informed consent to participate in this study was provided by the participants’ legal guardian/next of kin.

## Author Contributions

XX, SL, and QZ recruited patients. JY, LZ, WS, XX, and SL collected the clinical data. XX and QZ performed whole exome analysis. QZ, JY, and LZ performed the bioinformatic analysis and designed the study. JY, LZ, JO, WS, and QZ discussed the results and wrote the manuscript. All authors reviewed and approved the manuscript.

## Conflict of Interest

The authors declare that the research was conducted in the absence of any commercial or financial relationships that could be construed as a potential conflict of interest.

## Footnotes

1
http://genome.ucsc.edu/

2
http://bio-bwa.sourceforge.net/

3
http://samtools.sourceforge.net/

4
http://snpeff.sourceforge.net/

5
http://annovar.openbioinformatics.org/en/latest/

6
http://varianttools.sourceforge.net/Annotation/DbNSFP

7
https://cadd.gs.washington.edu/info

8
https://sites.google.com/site/revelgenomics/

9
http://gnomad.broadinstitute.org/

10
http://www.hgmd.cf.ac.uk/ac/index.php

11
https://www.ncbi.nlm.nih.gov/pubmed/

12
http://apps.webofknowledge.com/

13
https://scholar.google.com/

## Supplementary Material

Supplementary Figure 1 Pedigrees of 51 families with likely pathogenic variants in *RPGR*. Previously reported of *RPGR* variants identified in families by Sanger sequencing are not shown. The family ID is provided above each pedigree. The probands and available family members were analyzed by Sanger sequencing. Arrows, probands of each family; filled symbols, patients with different eye diseases; M, mutation; +, wild type; square, male; circle, female.

Supplementary Figure 2 Sanger sequencing of 51 unrelated families with likely pathogenic variants. Pedigrees are shown in the left column. Diagrams of the mutant sequence and the corresponding normal control sequence diagram are shown in the columns on the right. Sites of sequence changes are shown above the sequence and indicated by a black arrow.

Supplementary Figure 3 Pedigrees with likely benign variants. The family ID is provided above each pedigree. The probands and available family members were identified by Sanger sequencing. Arrows, probands of each family; filled symbols, patients with different eye diseases; M, mutation; +, wild type; square, male; circle, female.

Supplementary Figure 4 Comparison of phenotypes according to different factors in male patients. **(A–C)** Refractive error were not associated with location and variation type, there was no statistical significance. M + I, missense and in-frame; T, truncation; E1-14, exon1-exon14; ns, no statistical significance; RCC1, RCC1-like domain.

Supplementary Figure 5 Comparison of phenotypes according to different factors in female carriers. **(A–F)** The severity of BCVA and refractive error show no correlation with different location, variation type, there was no statistical significance. M + I, missense and in-frame; T, truncation; E1-14, exon1-exon14; BCVA, best corrected visual acuity; ns, no statistical significance; RCC1, RCC1-like domain.

## Abbreviations

AF: allele frequency
All: all population
BCVA: best corrected visual acuity
CD: corneal degeneration
COD: cone dystrophy
CORD: cone-rod dystrophy
DM: disease-causing mutations
EA: East Asians
G: glaucoma
HM: high myopia
Hyp: hypermetropia
IRD: inherited retinal degenerations
LCA: Leber congenital amaurosis
LD: lens dislocation
MC: macular coloboma
MD: macular degeneration
N: normal
NA: not available
NYS: nystagmus
OA: ocular albinism
ONH: Optic nerve hypoplasia
PHPV: Persistent Hyperplastic Primary Vitreous
RB: retinoblastoma
RD: retinal diseases
RE: refractive error
RP: retinitis pigmentosa
RRD: Rhegmatogenous Retinal Detachment
SA: splicing acceptor
SD: splicing donor
SER: spherical equivalent refraction. BDGP Berkeley Drosophila Genome Project
gnomAD: genome aggregation database
HGMD: the Human Gene Mutation Database
HSF: Human Splicing Finder.
