# Supplementary figures and images for: Genotype–Phenotype Analysis of RPGR Variations: Reporting of 62 Chinese Families and a Literature Review
Source: Front Genet. 2021 Jun 23;12:600210. doi: 10.3389/fgene.2021.600210 (PMC8565807; doi:10.3389/fgene.2021.600210)

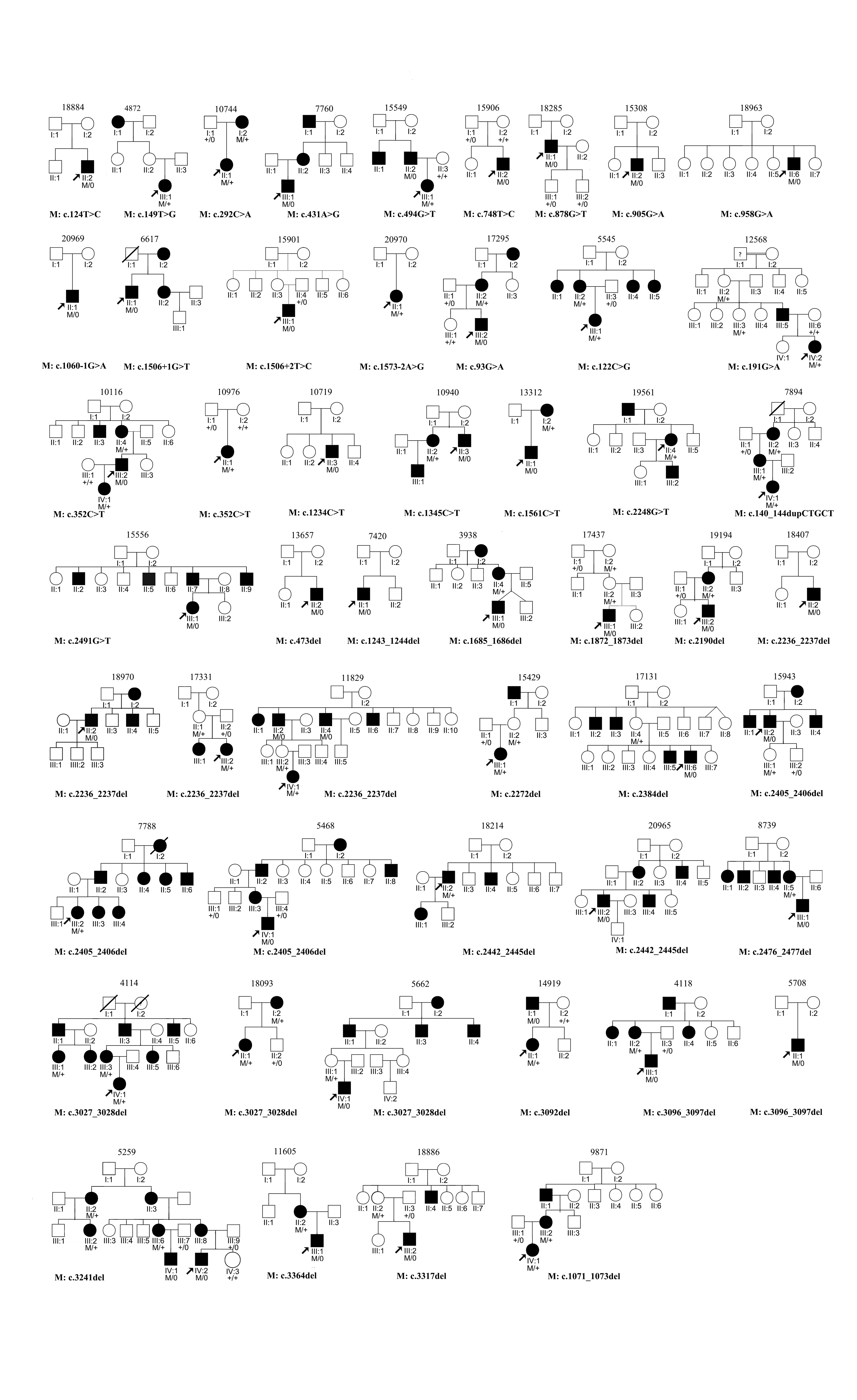

Supplement: Supplementary Figure 1 — Pedigrees of 51 families with likely pathogenic variants in RPGR. Previously reported of RPGR variants identified in families by Sanger sequencing are not shown. The family ID is provided above each pedigree. The probands and available family members were analyzed by Sanger sequencing. Arrows, probands of each family; filled symbols, patients with different eye diseases; M, mutation; +, wild type; square, male; circle, female. [file Image_1.TIF]

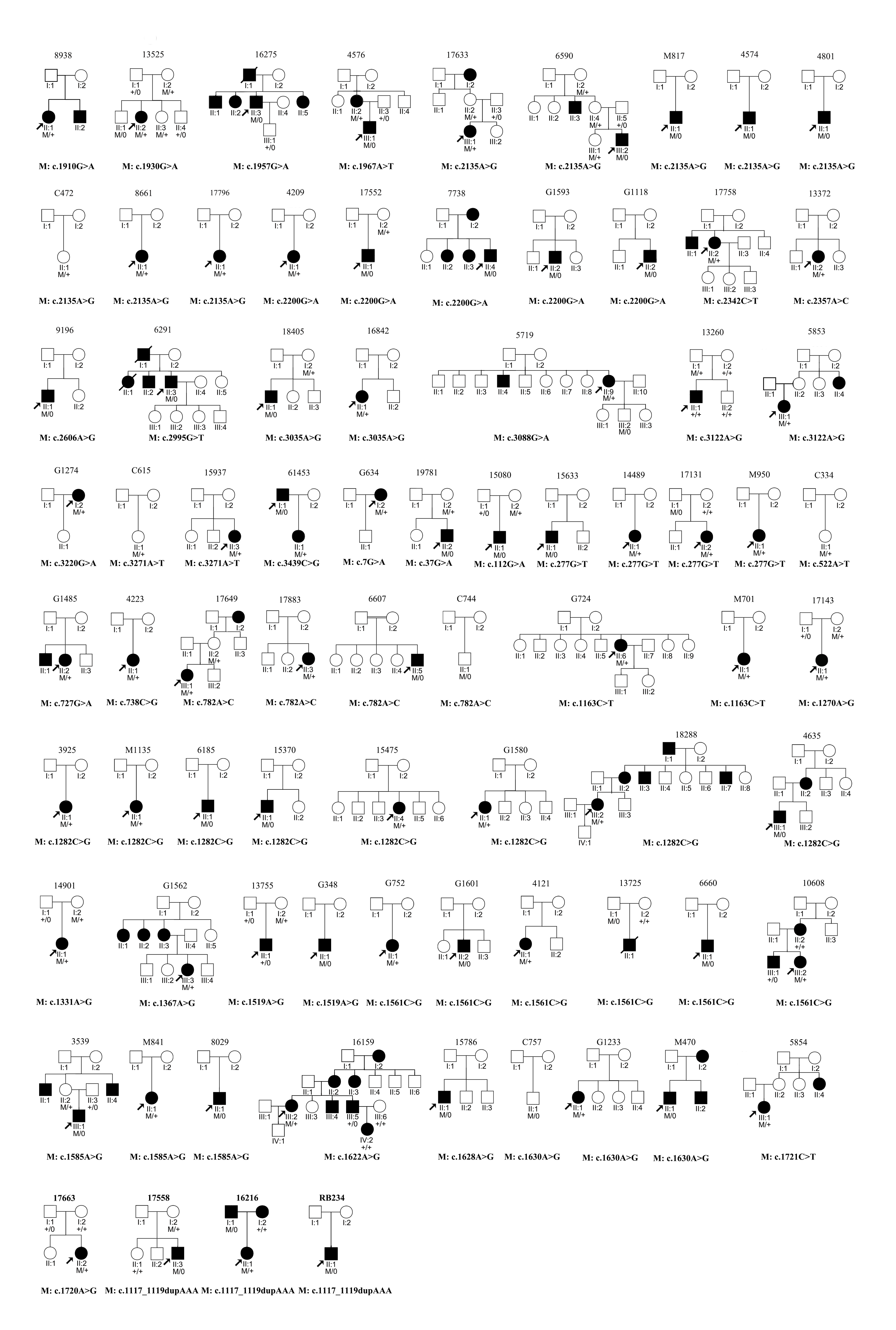

Supplement: Supplementary Figure 2 — Sanger sequencing of 51 unrelated families with likely pathogenic variants. Pedigrees are shown in the left column. Diagrams of the mutant sequence and the corresponding normal control sequence diagram are shown in the columns on the right. Sites of sequence changes are shown above the sequence and indicated by a black arrow. [file Image_2.TIF]

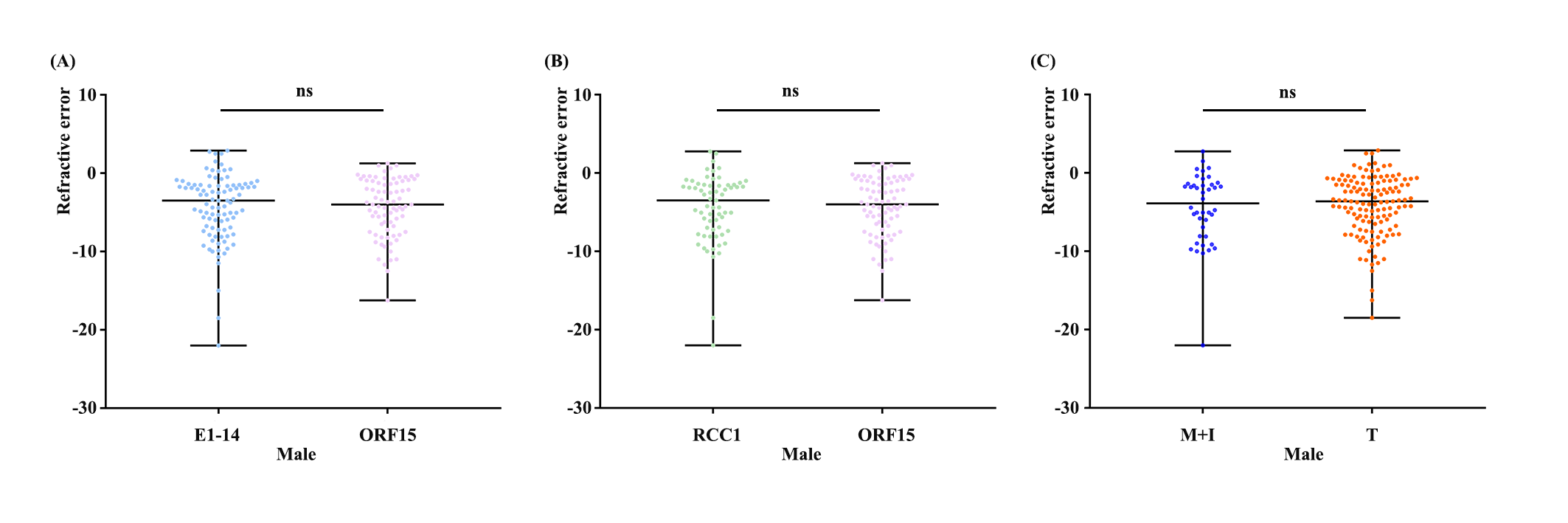

Supplement: Supplementary Figure 3 — Pedigrees with likely benign variants. The family ID is provided above each pedigree. The probands and available family members were identified by Sanger sequencing. Arrows, probands of each family; filled symbols, patients with different eye diseases; M, mutation; +, wild type; square, male; circle, female. [file Image_3.TIF]

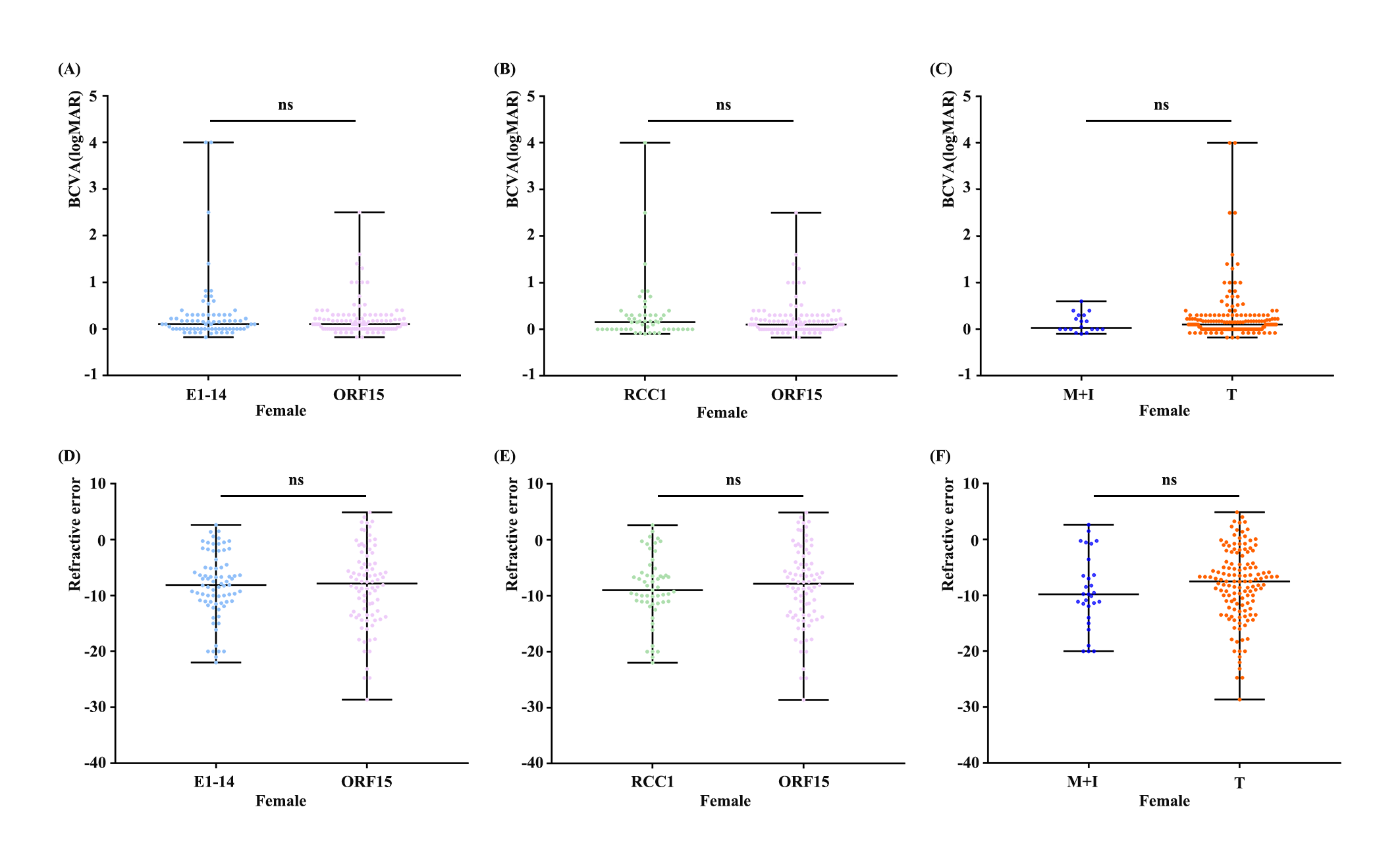

Supplement: Supplementary Figure 4 — Comparison of phenotypes according to different factors in male patients. (A–C) Refractive error were not associated with location and variation type, there was no statistical significance. M + I, missense and in-frame; T, truncation; E1-14, exon1-exon14; ns, no statistical significance; RCC1, RCC1-like domain. [file Image_4.TIF]

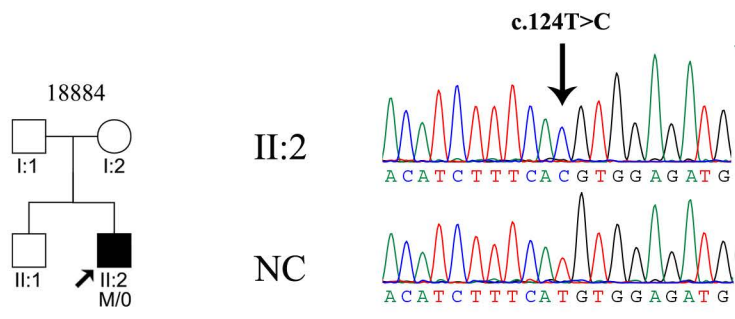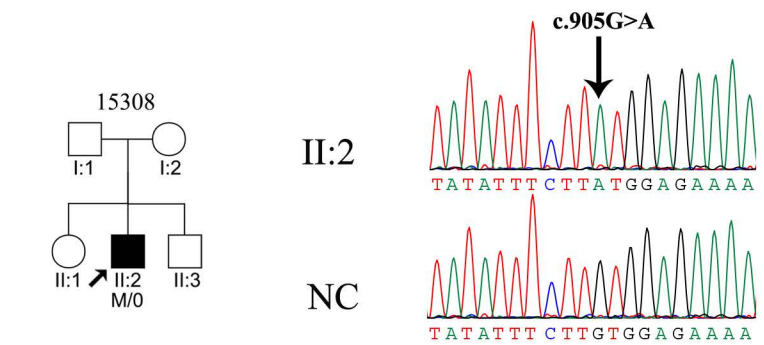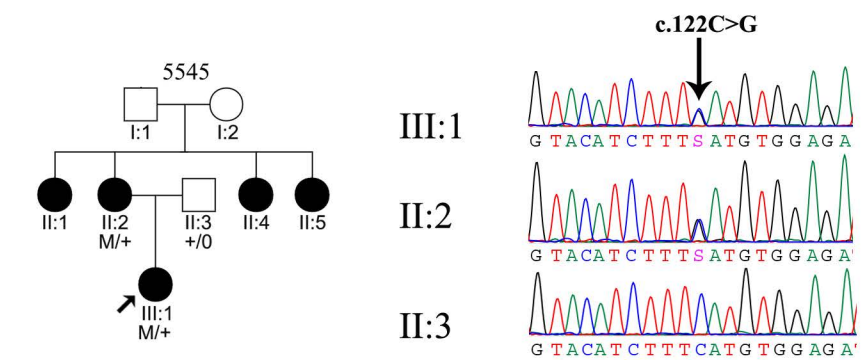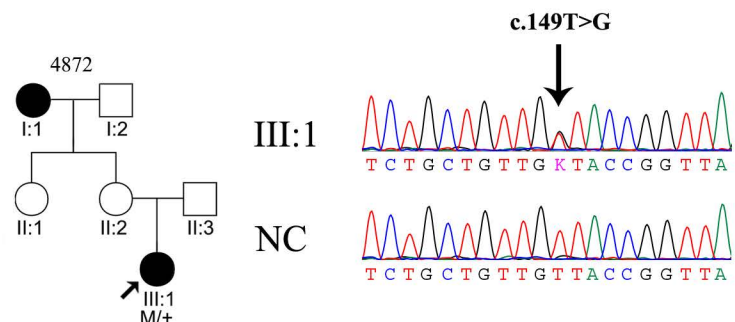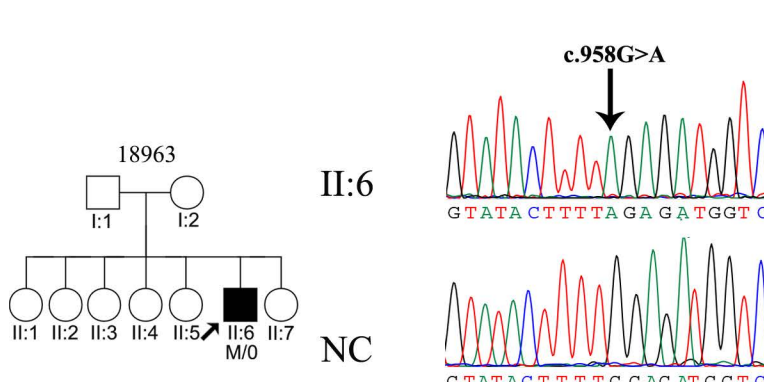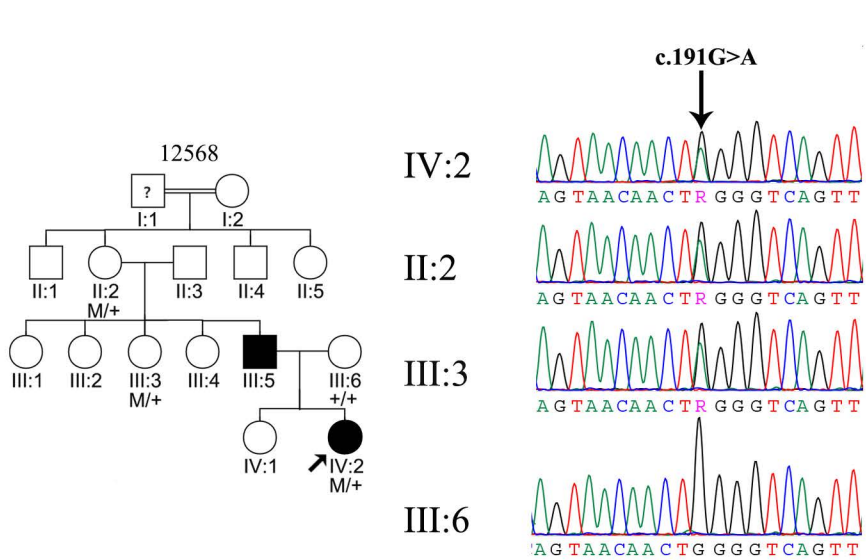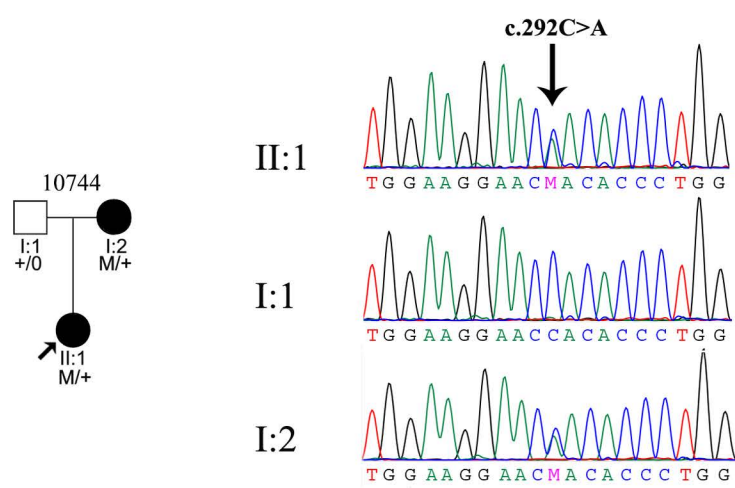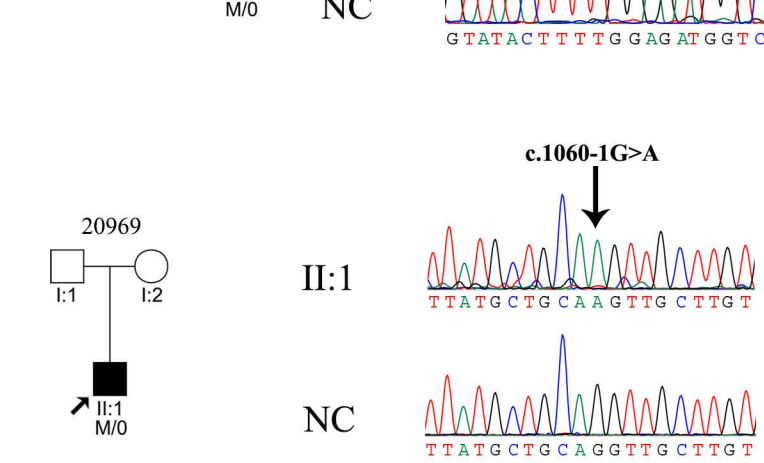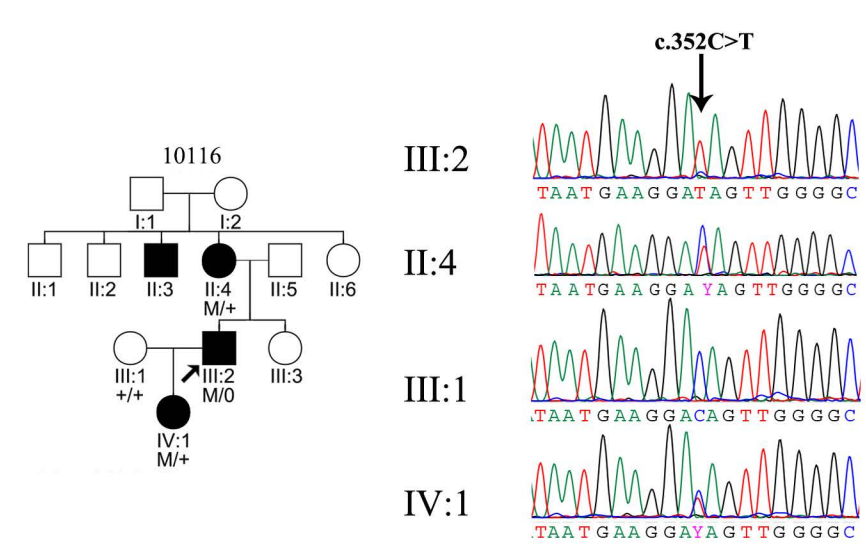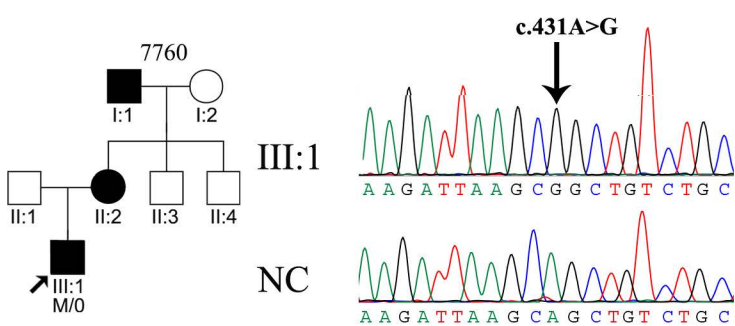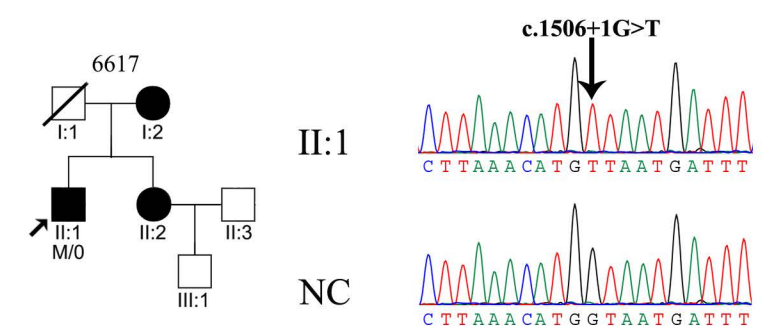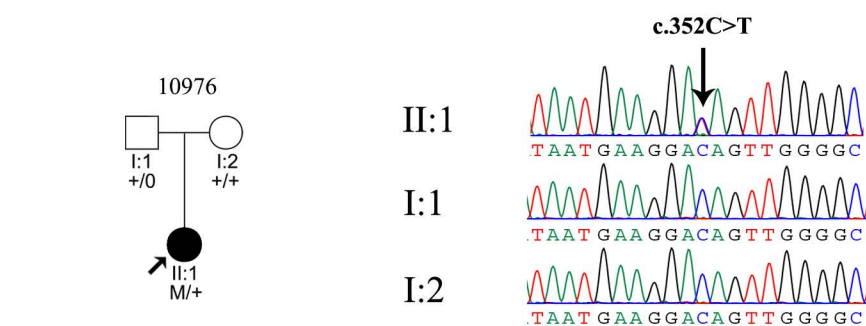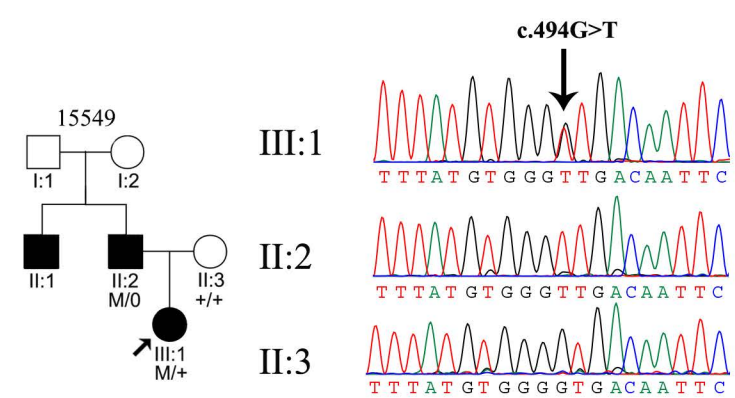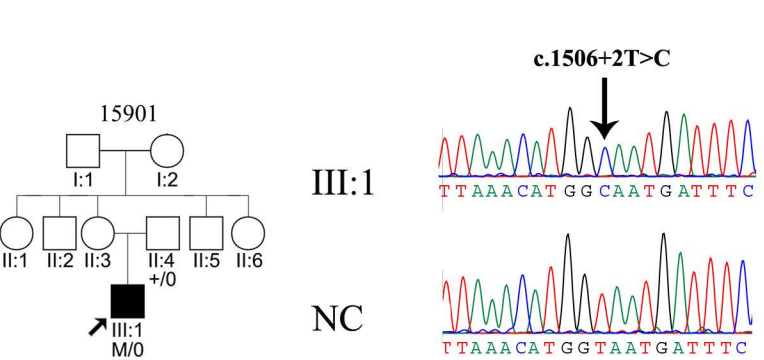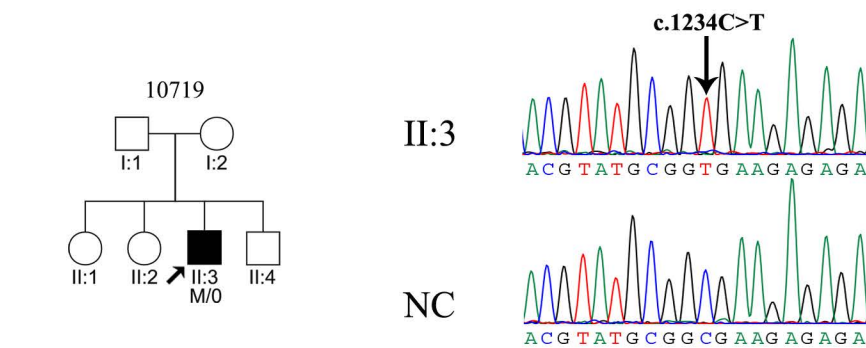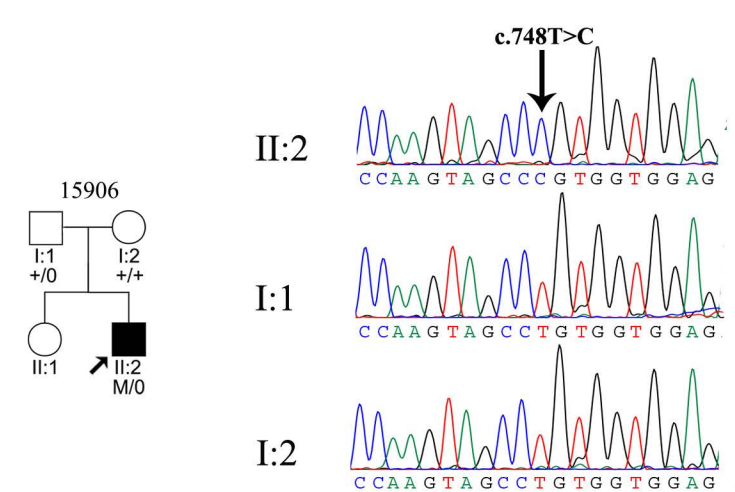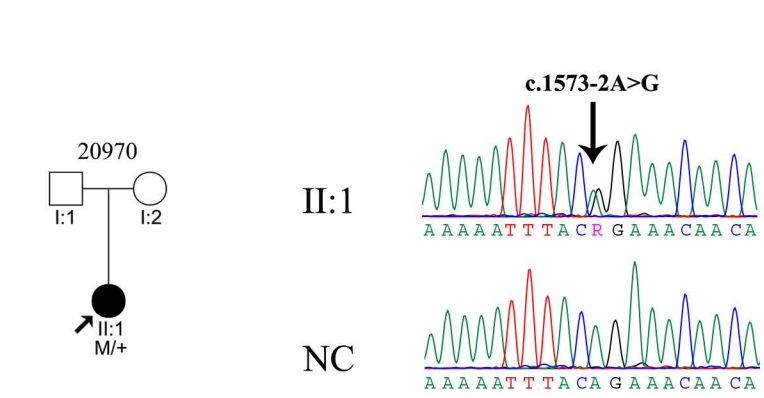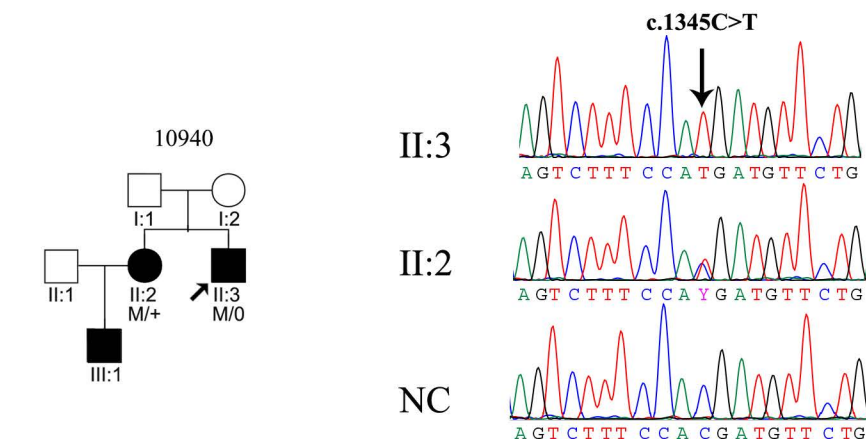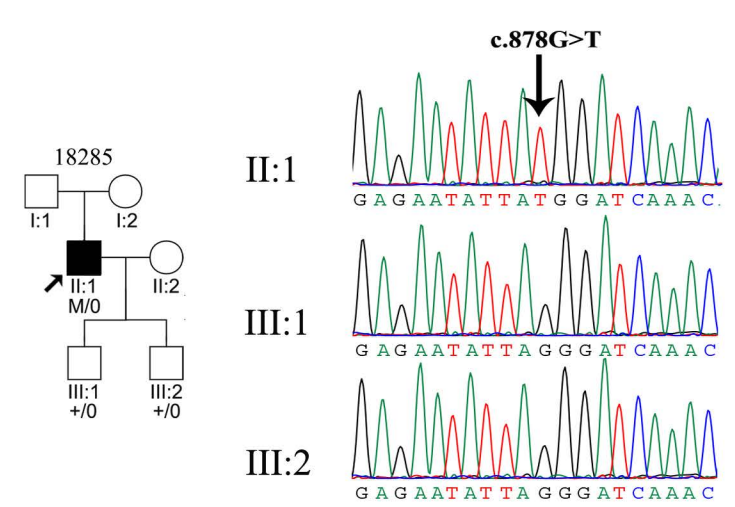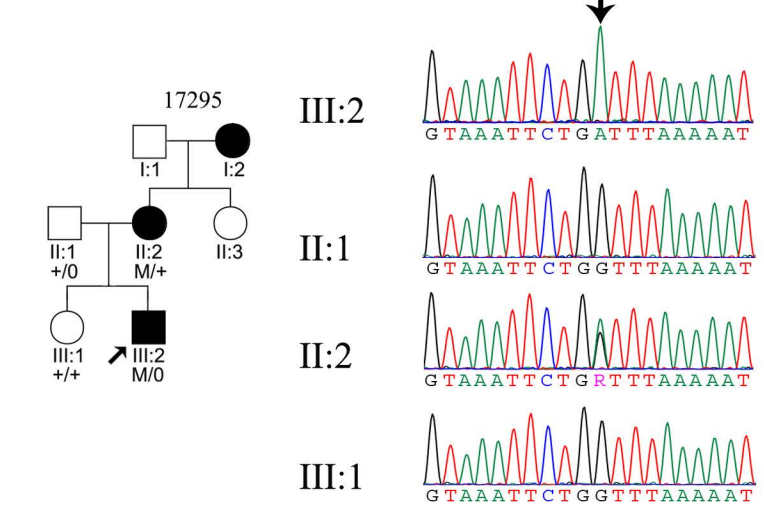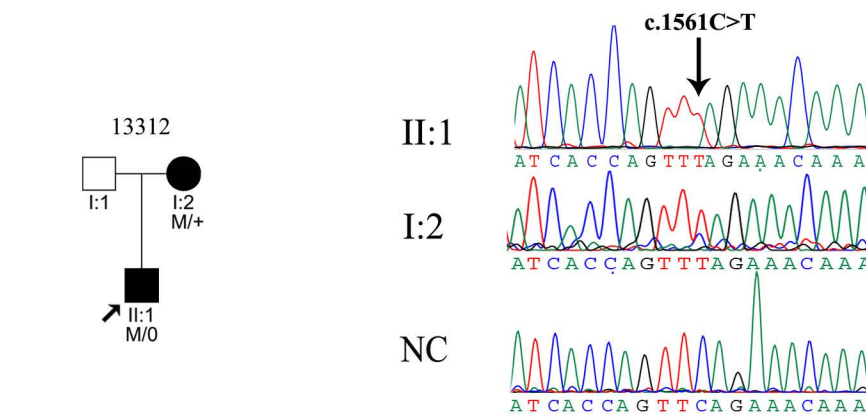

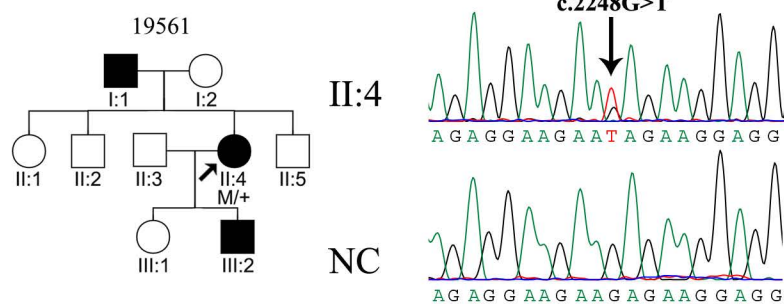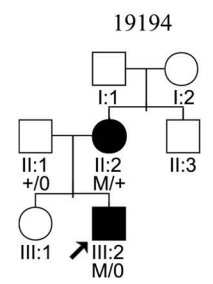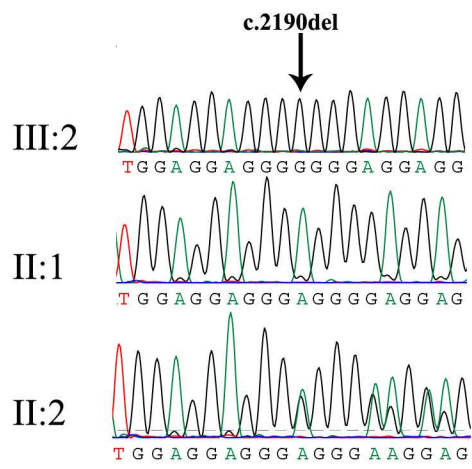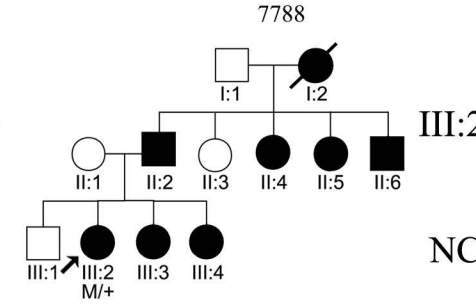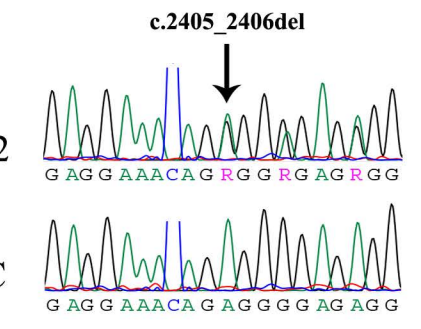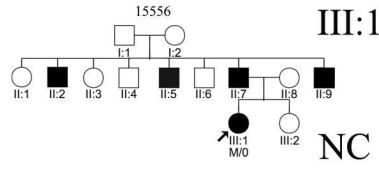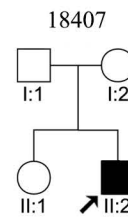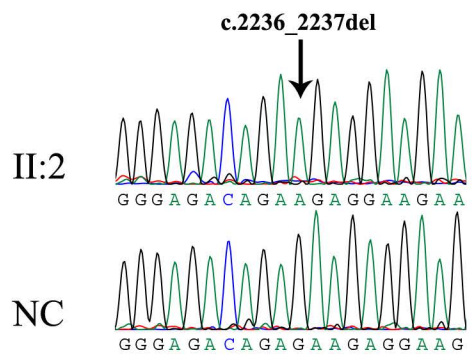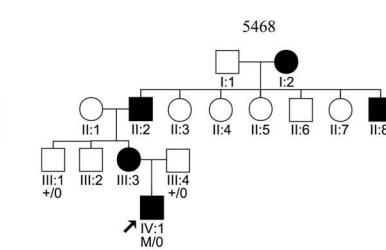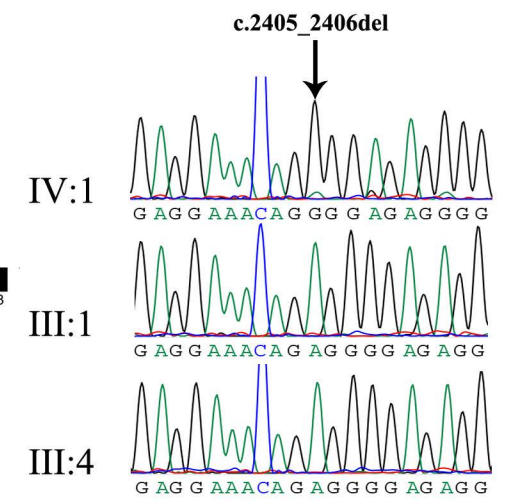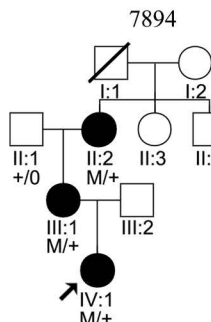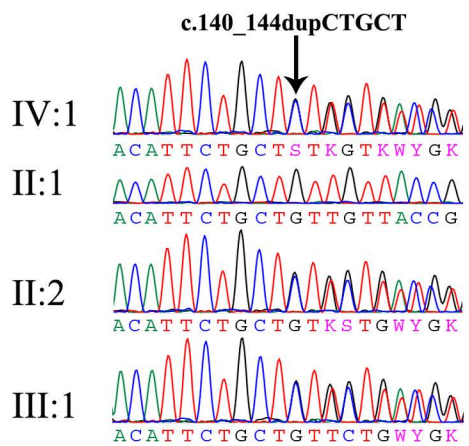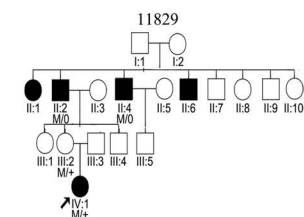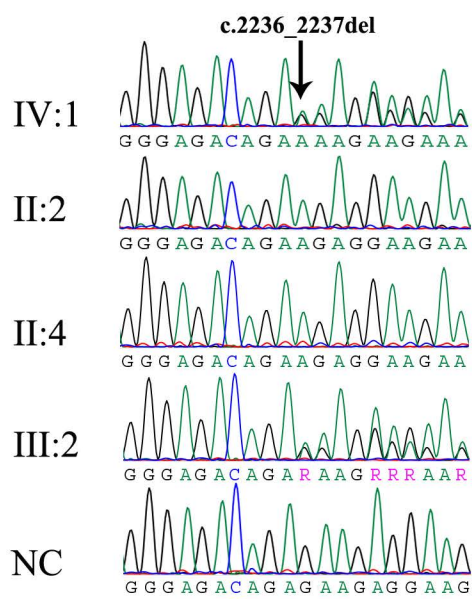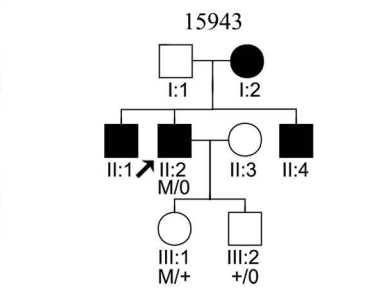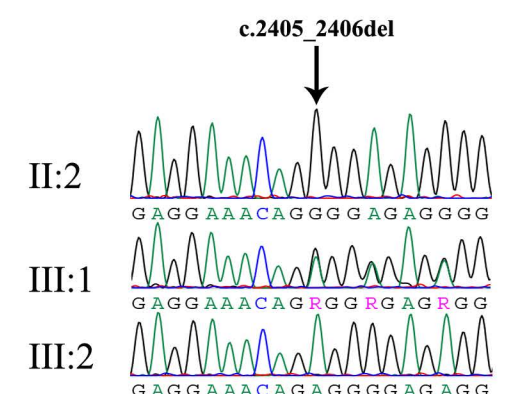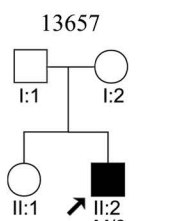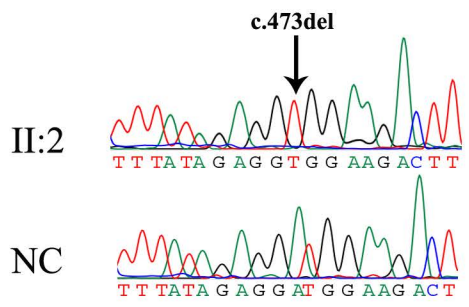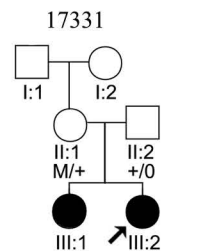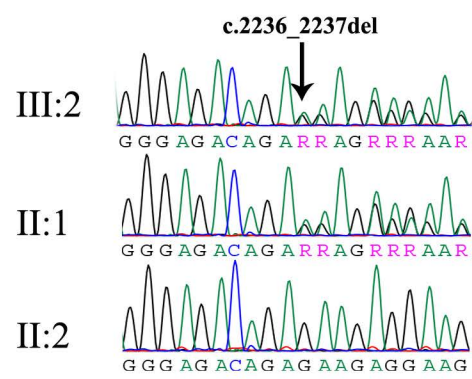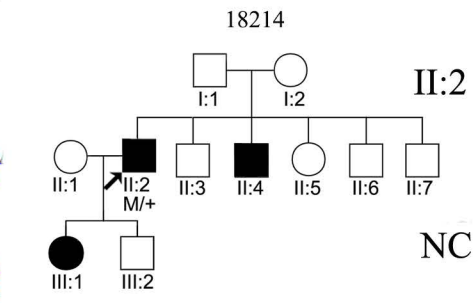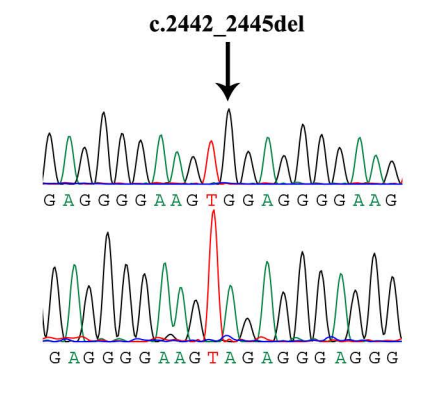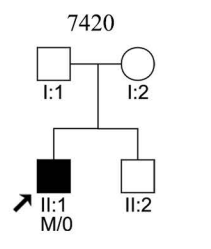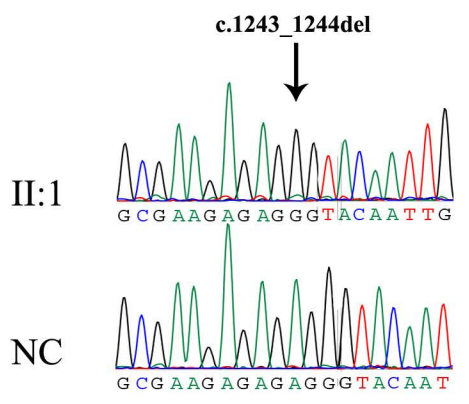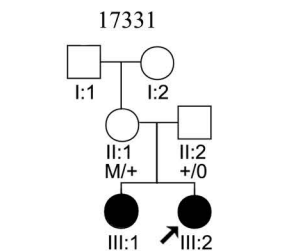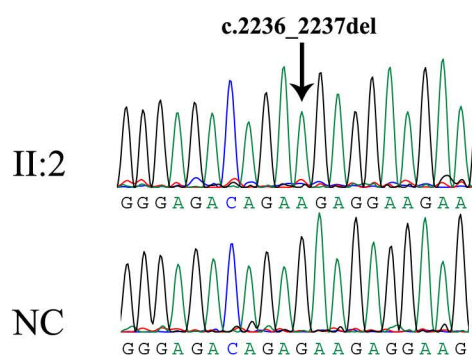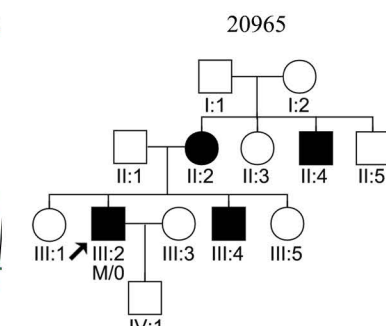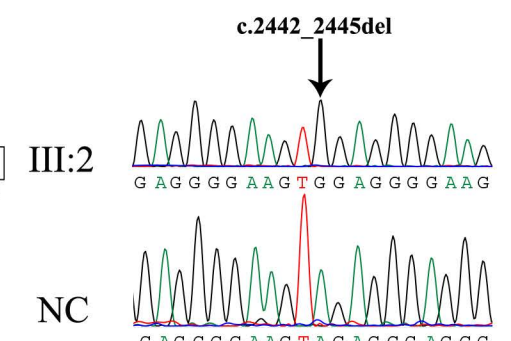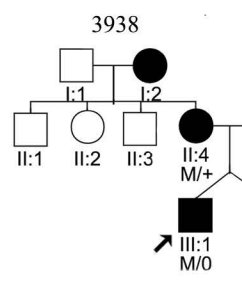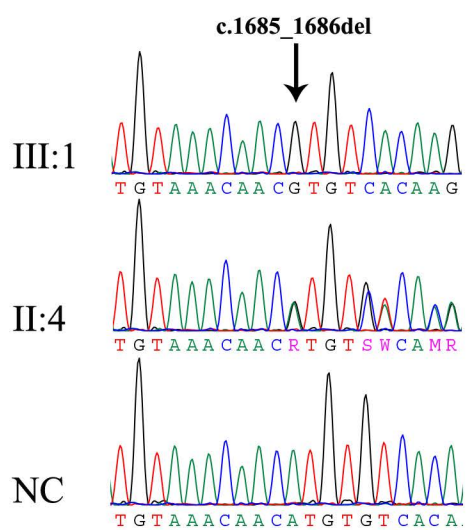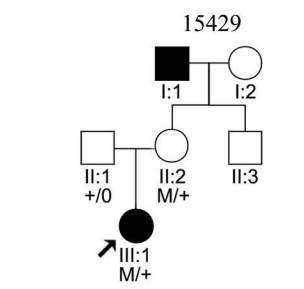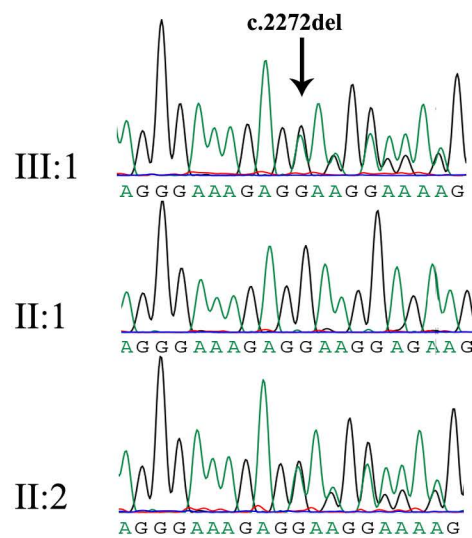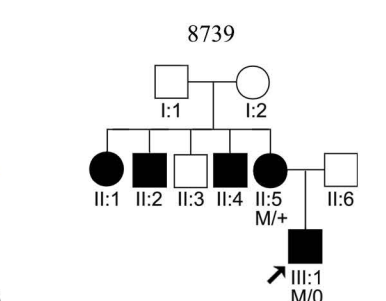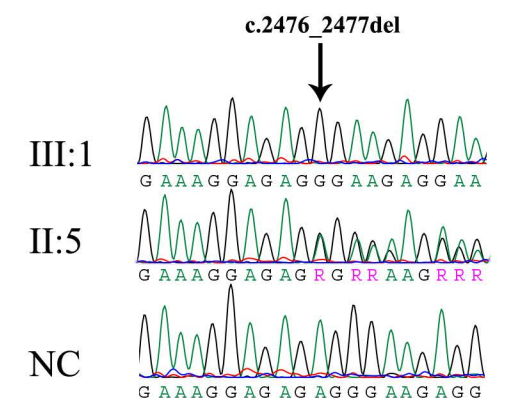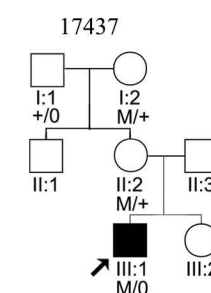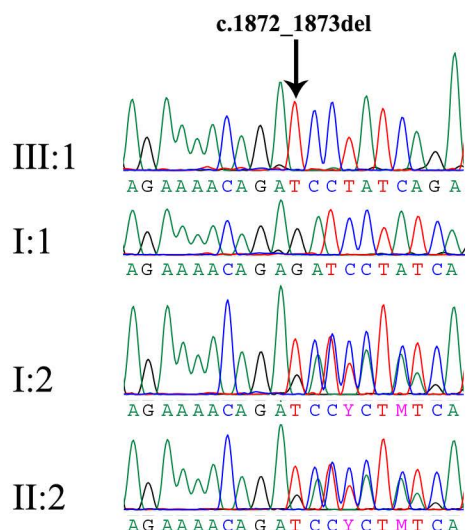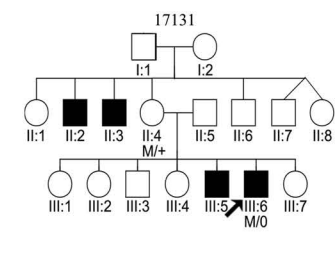

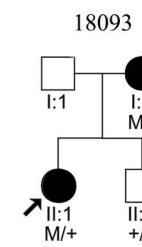

II:1

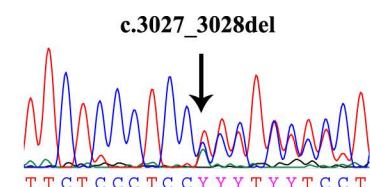

I:2

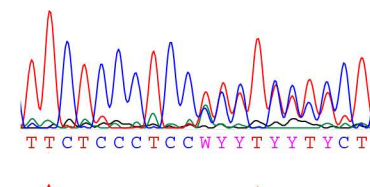

II:2

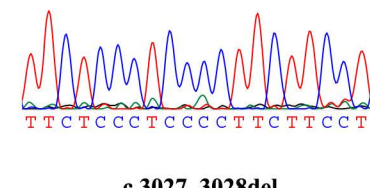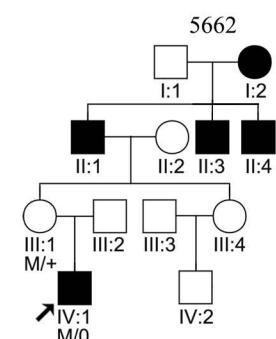

IV:1

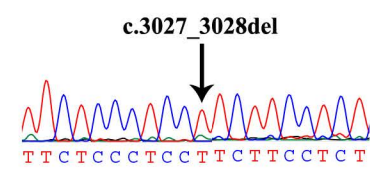

III:1

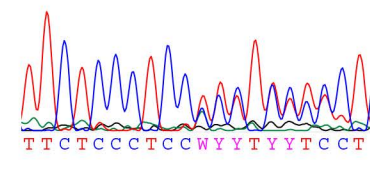

NC

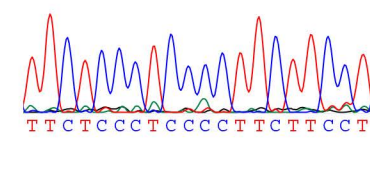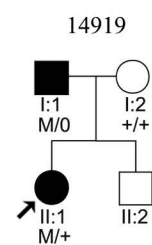

II:1

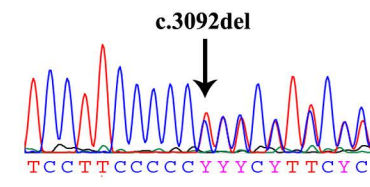

I:1

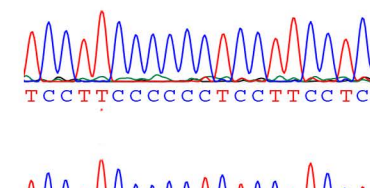

I:2

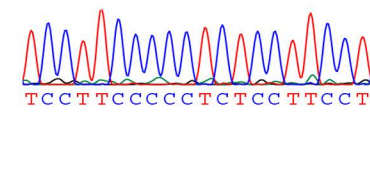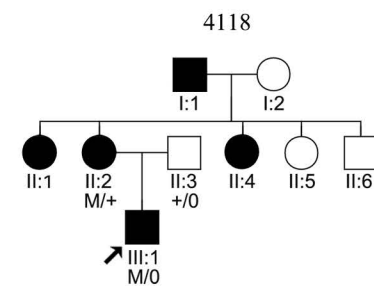

III:1

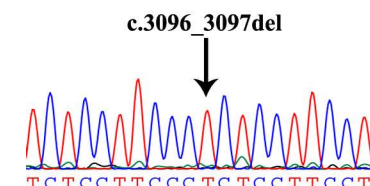

II:2

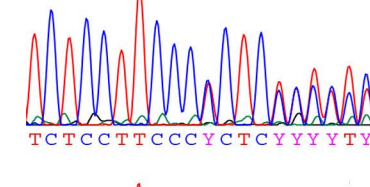

II:3

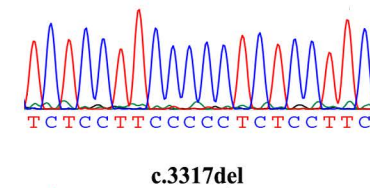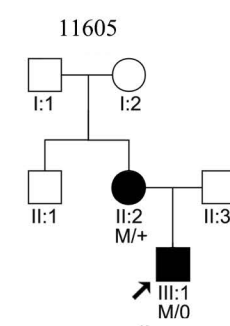

III:1

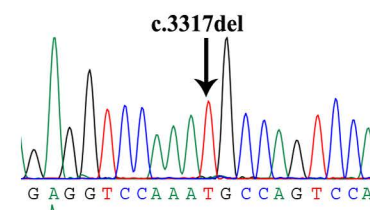

II:2

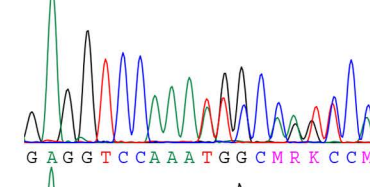

NC

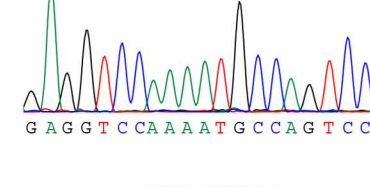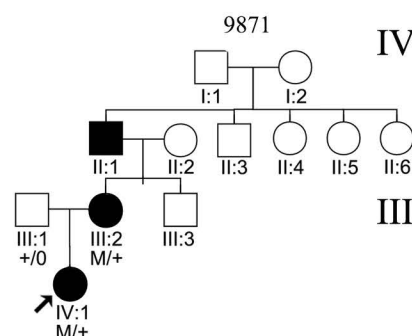

IV:1

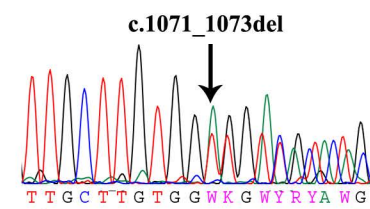

III:1

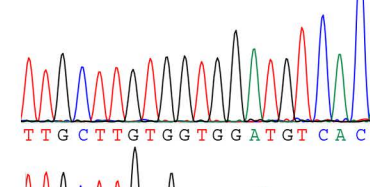

III:2

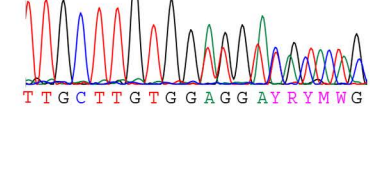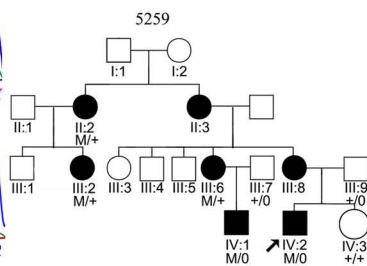

IV:2

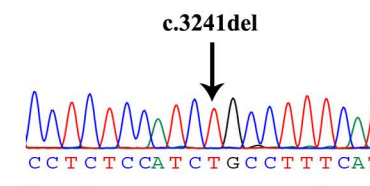

II:2

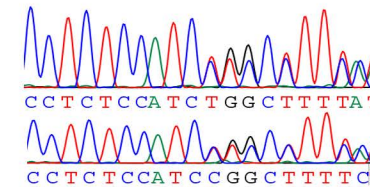

III:2

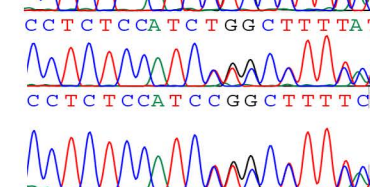

III:6

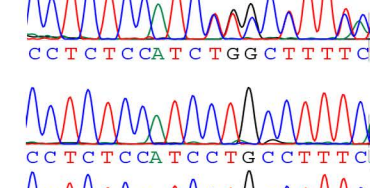

III:7

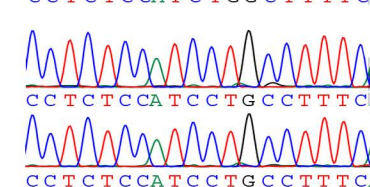

III:9

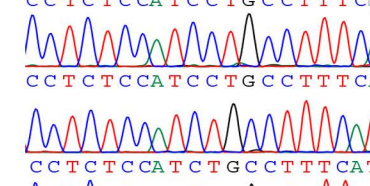

IV:1

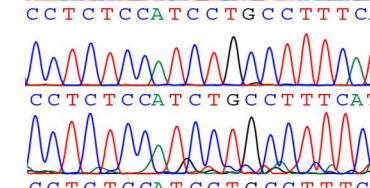

IV:3

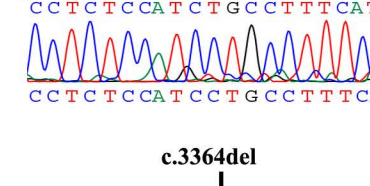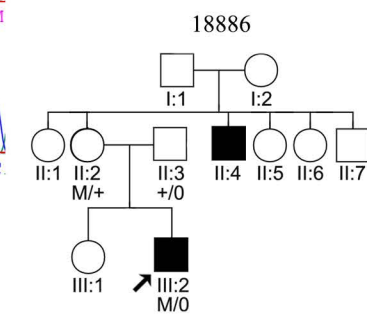

III:2

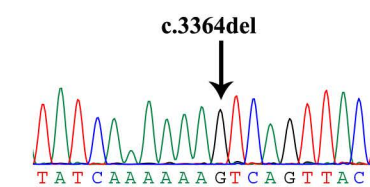

II:2

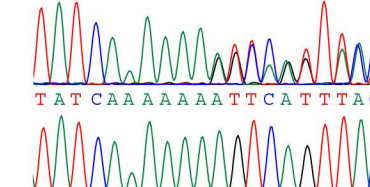

II:3

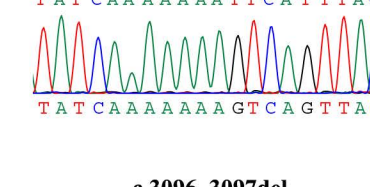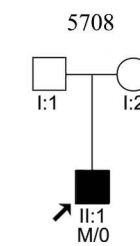

II:1

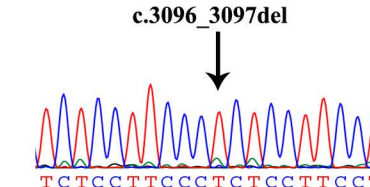

NC

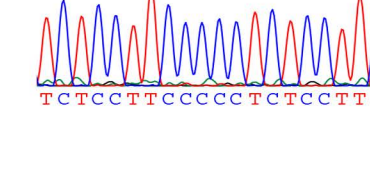

Supplement: Supplementary file 5 [file Data_Sheet_1.PDF]

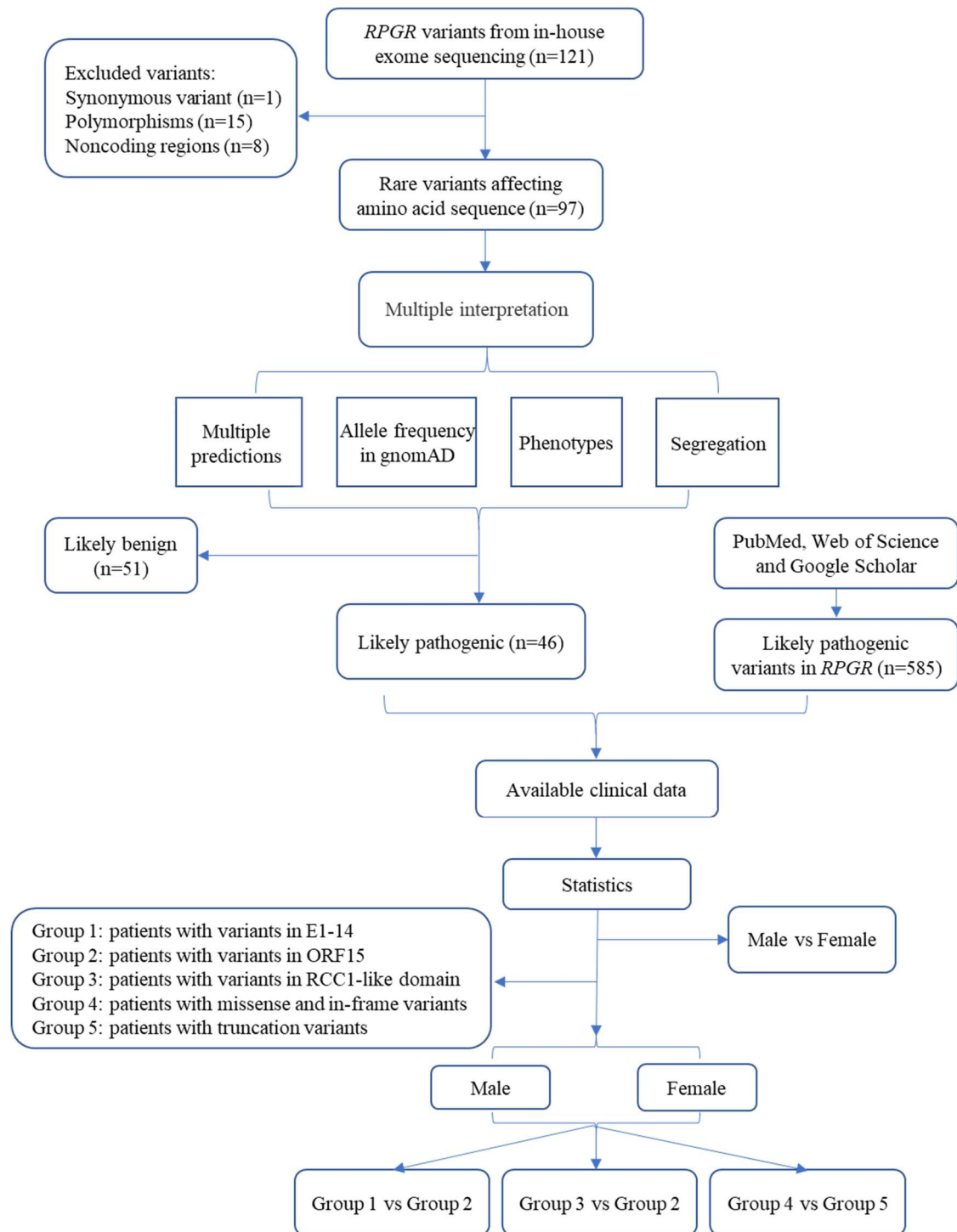

Flowchart of the data analysis

Supplement: Supplementary Figure 5 — Comparison of phenotypes according to different factors in female carriers. (A–F) The severity of BCVA and refractive error show no correlation with different location, variation type, there was no statistical significance. M + I, missense and in-frame; T, truncation; E1-14, exon1-exon14; BCVA, best corrected visual acuity; ns, no statistical significance; RCC1, RCC1-like domain. [file Data_Sheet_2.PDF]
